# Modelling mutational and selection pressures on dinucleotides in eukaryotic phyla –selection against CpG and UpA in cytoplasmically expressed RNA and in RNA viruses

**DOI:** 10.1186/1471-2164-14-610

**Published:** 2013-09-10

**Authors:** Peter Simmonds, Wenjun Xia, J Kenneth Baillie, Ken McKinnon

**Affiliations:** 1 Division of Infection and Immunity, Roslin Institute, University of Edinburgh, Easter Bush, Edinburgh EH25 9RG, UK; 2 School of Mathematics, University of Edinburgh, James Clerk Maxwell Building, The King’s Buildings, Edinburgh EH9 3JZ, UK; 3 Division of Genetics and Genomics and Roslin Institute, University of Edinburgh, Easter Bush, Edinburgh EH25 9RG, UK

**Keywords:** Dinucleotide, Methylation, Exon, RNA virus, Markov modelling

## Abstract

**Background:**

Loss of CpG dinucleotides in genomic DNA through methylation-induced mutation is characteristic of vertebrates and plants. However, these and other eukaryotic phyla show a range of other dinucleotide frequency biases with currently uncharacterized underlying mutational or selection mechanisms. We developed a parameterized Markov process to identify what neighbour context-dependent mutations best accounted for patterns of dinucleotide frequency biases in genomic and cytoplasmically expressed mRNA sequences of different vertebrates, other eukaryotic groups and RNA viruses that infect them.

**Results:**

Consistently, 11- to 14-fold greater frequencies of the methylation-associated mutation of C to T upstream of G (depicted as C→T,G) than other transitions best modelled dinucleotide frequencies in mammalian genomic DNA. However, further mutations such as G→T,T (5-fold greater than the default transversion rate) were required to account for the full spectrum of dinucleotide frequencies in mammalian sequence datasets. Consistent with modeling predictions for these two mutations, instability of both CpG and CpT dinucleotides was identified through SNP frequency analysis of human DNA sequences. Different sets of context-dependent mutations were modelled in other eukaryotes with non-methylated genomic DNA. In contrast to genomic DNA, best-fit models of dinucleotide frequencies in transcribed RNA sequences expressed in the cytoplasm from all organisms were dominated by mutations that eliminated UpA dinucleotides, observations consistent with cytoplasmically driven selection for mRNA stability. Surprisingly, mRNA sequences from organisms with methylated genomes showed evidence for additional selection against CpG through further context-dependent mutations (eg. C→A,G). Similar mutation or selection processes were identified among single-stranded mammalian RNA viruses; these potentially account for their previously described but unexplained under-representations of CpG and UpA dinucleotides.

**Conclusions:**

Methods we have developed identify mutational processes and selection pressures in organisms that provide new insights into nucleotide compositional constraints and a wealth of biochemical and evolutionarily testable predictions for the future.

## Background

One of the most striking compositional abnormalities in DNA sequences of mammalian and other vertebrate genomic DNA sequences is the marked under-representation of CpG and over-representation of CpA and TpG dinucleotides. This compositional abnormality was first recognized over 50 years ago [1-3] and is now generally accepted to result directly from the mutagenic effect of methylation of cytosine (mC) bases in CpG dinucleotides. mC is more likely to deaminate to thymine [4,5] so depleting CpG dinucleotides and increasing the frequencies of TpG and CpA on the opposite strand through mismatch repair.

In more general terms, dinucleotide compositional abnormalities reflect either context-sensitive differences in mutation rates (as in the case of DNA methylation and CpG under-representation) or specific selection for or against certain dinucleotides. As an example of the latter, the UpA dinucleotide is targeted by RNA-degrading enzymes and its presence in an RNA sequence accelerates its degradation in the cytoplasm. UpA composition therefore modulates protein expression from mRNA through its influence on transcriptome turnover [6,7]. The widespread suppression of UpA dinucleotides in mRNA sequences may therefore reflect selection for increased stability in the cytoplasm.

In spite of these two well known examples, it remains unclear whether the combination of mutational biases against CpG in genomic DNA and selection against UpA in mRNA accounts for the complex pattern of over- and under-representation of each of the 16 dinucleotides in vertebrates. Secondly it remains unexplained why the degree of CpG under-representation is inversely proportional to the G+C content of the underlying sequence, although it has been speculated that there are differences in the accessibility of genomic DNA in high and low G+C regions to deamination [8,9]. Thirdly, the observation made many years ago that many RNA viruses under-represent CpG dinucleotides despite the absence of a specific (methylation-dependent) mutational pathway for RNA has remained unexplained [10-12]. Patterns of CpG and UpA under-representation among viruses infecting hosts with different degrees of host genomic DNA methylation have remained similarly unexplored. Finally, eukaryotes with non-methylated genomes show different patterns of dinucleotide representation (such as elevated frequencies of ApA in ecdysozoa) for which neither a mutational nor a selectionist mechanism has yet been proposed.

Using data from a range of eukaryotes with different methylation patterns, Simmen showed that the degree of over-representation of CpA and TpG dinucleotides were in proportion to the expected frequency created by C→T transitions in methylated DNA [13]. In Duret and Galtier [14], an explicit mathematical model was developed to investigate whether frequent CpG-context dependent mutations could account for the suppression in frequencies of TpA in human DNA sequences. Assignment of an elevated C→T transition rate reproduced the CpG deficit (and G+C dependence) observed in mammalian DNA and indirectly depleted TpA dinucleotide frequencies. However, this model failed to account for the full extent of TpA depletion observed in human DNA sequences and the model was not applied to investigate the effect of this single mutational bias on other dinucleotide frequencies, such as TpG and CpA that also show compositional biases. How well this model might fully recreate the dinucleotide profile of human DNA remains unresolved.

In the current study we have developed an extended model of sequence evolution that allows separate mutation rates for each type of transition and transversion in each dinucleotide context against a background, separately optimized mean transition / transversion ratio (*κ*). This model generalizes Duret and Galtier’s model [14], in which *κ* was fixed at 2.1 and only one context dependent mutation, (C→T,G) was allowed to take a higher mutation rate. (This rate was based on observational data available at the time of the study on sequence variability in human DNA sequences.) Our approach in contrast allowed up to 48 (or 96 for RNA) different dinucleotide context dependent mutations and optimised rates to maximise the fit between model predictions and observed frequencies of all 16 dinucleotides. In the specific case of analysing human DNA, the mutation C→T, G and a transition rate of around 12 were discovered by the modelling rather than being imposed *a priori.* This analysis was also extended to the corresponding mRNA sequences to investigate whether additional or different mutational or selection pressures were exerted in cytoplasmically expressed sequences.

Modelling was extended to other mammalian DNA and mRNA datasets, organisms showing largely absent genomic DNA methylation (fish, insects, nematodes) and mammalian RNA viruses in which the phenomenon of CpG under-representation has been previously described [10,11]. Modelling was naturally restricted to processes showing global effects on DNA composition and was unsuited for modelling effects of genome modifications with specific functional roles. The latter include the recently discovered role of DNA methylation in the gene expression and development pathways of the honey bee (*Apis mellifera*) and other insects [15,16] that possess primarily non-methylated genomes. Modelling was also restricted to mutational processes or selection operating in dinucleotide contexts. While methylation (and associated mutations) primarily occurs in a CpG context in vertebrates and where studied in other eukaryotic groups, plant genomes are additionally heavily methylated (50-80%) in the CpA/T/CpG trinucleotide context [17]. As this potentially exerts a significant additional mutational pressure on plant genomic DNA and cannot be modelled, the analysis of plant genome sequences was excluded from the current study.

On the larger genomic scale, we obtained evidence both for mutational processes acting on genomic DNA beyond simple methylation-induced hypermutation and for a range of additional likely selection pressures on mRNA that centre around the elimination of UpA dinucleotides. The existence of this selection pressure and its occurrence in RNA viruses provides evidence for a series of novel compositional constraints in the cytoplasm on viral RNA. Specific dinucleotides may be selected against to escape currently uncharacterized self/non-self recognition mechanisms that are coupled to the interferon system (in mammals) and potential parallel defence mechanisms in other eukaryotic phyla.

## Results

### Patterns of dinucleotide frequencies in genomic DNA and mRNA

Ratios of observed to expected frequencies of dinucleotides were computed for DNA genomic sequences of several different eukaryotes and their corresponding mRNA sequences. DNA datasets were restricted to sequences that were non-transcribed since mRNA sequences encoded by genomic DNA that enter the cytoplasm may be subject to additional selection pressures. These represent a relatively small component of mammalian DNA sequences (*H. sapiens*, *P. troglodytes* and *M. musculus* in the current study; 1.2-2.0%) but the proportion of cytoplasmically expressed sequences was much larger in other vertebrates and other eukaryotic phyla (6.5% - 28%).

**Figure 1:**
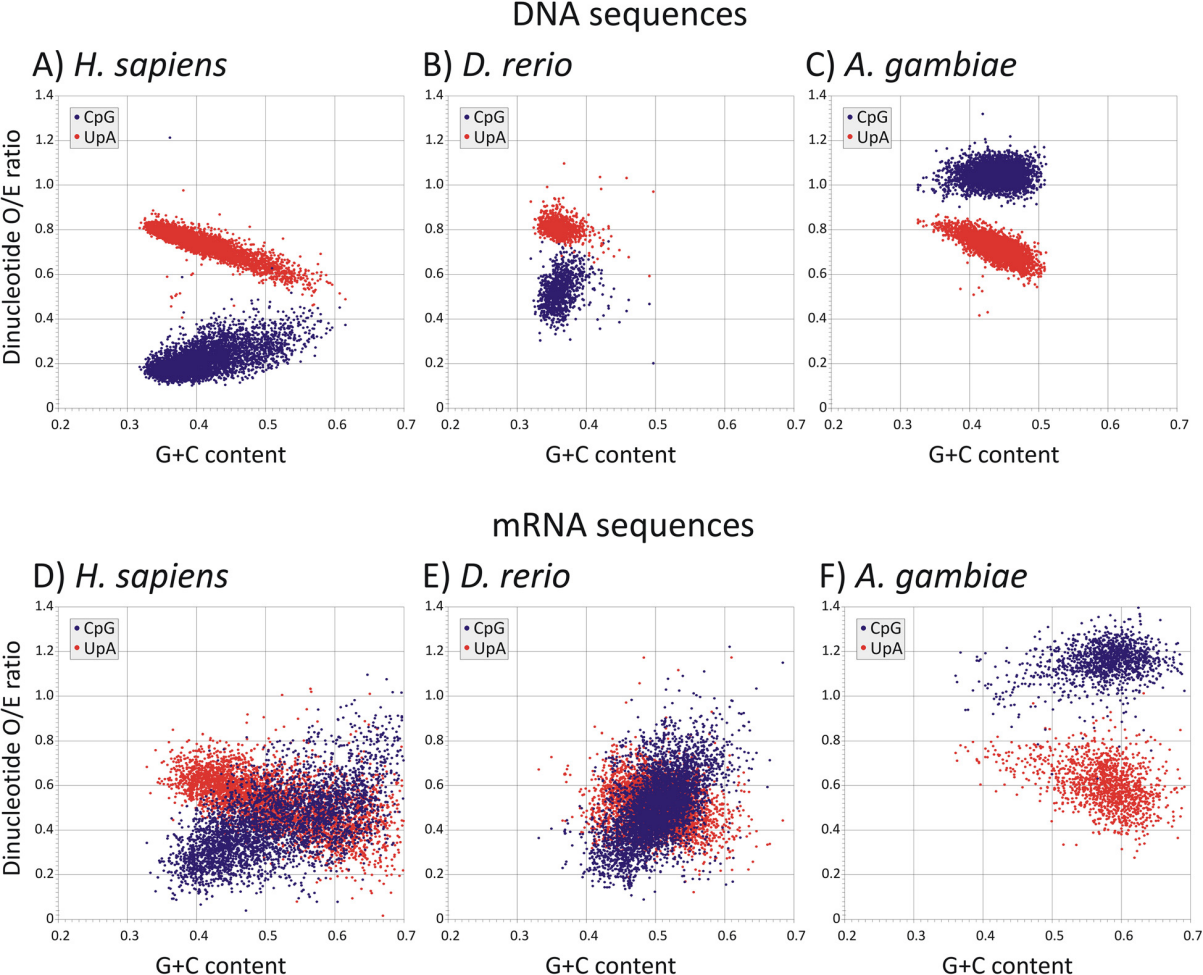
**G+C composition (x-axis) and frequencies of CpG and TpA (or UpA) dinucleotides in representative organisms with methylated (H. sapiens, D. rerio) and non-methylated genomes (A. gambiae), labelled in panels.** Symbols for CpG and UpA dinucleotide frequencies (blue and red dots respectively; see inset box) and were expressed as the ratio of observed frequency / frequency expected from mononucleotide (base) composition of the fragment.

As anticipated, frequencies of CpG dinucleotides in non-transcribed genomic DNA sequences from eukaryotic genomes showing extensive methylation (*H. sapiens, and D. rerio* [zebra fish]; Figures 1A, 1B) were substantially lower than expected from their G+C content. No such reduction was evident in DNA sequences of the mosquito, *A. gambiae* (Figure 1C) whose genome is largely unmethylated. Results from other mammals (*P. troglodytes* and *M. musculus*) were in practical terms identical to human DNA sequences) while other organisms without methylation of genomic DNA sequences (*Caenorhabditis elegans* [a nematode]*, Drosophila melanogaster* [fruit fly]) showed no under-representation of CpG dinucleotides (data not shown).

G+C contents of the subset of mRNA sequences were higher than non-cytoplasmically expressed sequences (Figure 1D, 1E, 1F). Several further differences between DNA and mRNA sequences were apparent in their dinucleotide compositions and their relationship with G+C composition. For example, for sequences with a given G+C content, UpA under-representation was greater in mRNA sequences than genomic sequences of humans (Figure 1A, 1D; *p* < 10^-10^ by Student t-test (Additional file 1: Table S1)). Even more evidently, UpA frequencies followed a quite different relationship with G+C content in *A. gambiae* and CpG frequencies in mRNA were substantially higher than in genomic DNA (Figure 1C, 1F). These observations are consistent with the existence of additional selection pressures on the subset of sequences expressed as mRNAs.

**Figure 2:**
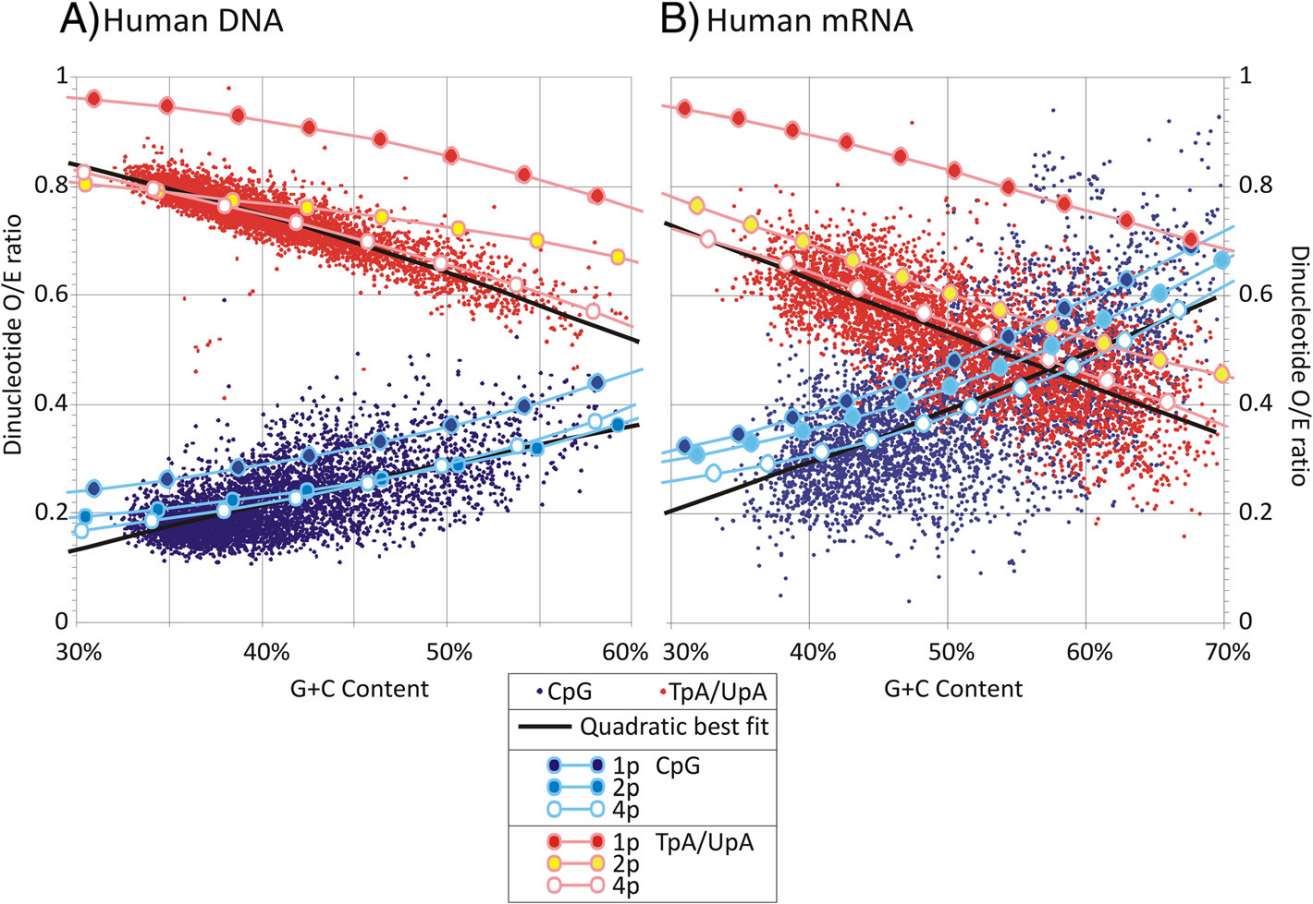
**Observed / expected CpG and UpA frequencies in (A) human DNA and (B) mRNA sequences as a function of G+C content.** Frequencies of each dinucleotide predicted from mutational models with 1, 2 and 4 parameters (1p, 2p and 4p, labelled according to the inset box) were superimposed on observed distributions of CpG and UpA dinucleotides (blue and red dots respectively; see inset box). Quadratic lines of best fit through observed distribution (black lines) were matched to model predictions over a G+C composition range from 20%-80%.

Compositional biases extended to other dinucleotides in humans (Figure 2A, 2B) and other organisms (Additional file 2: Figure S1). Several instances of compositional asymmetries are evident in complementary dinucleotides in mRNA sequences, such as the higher frequencies of UpC dinucleotides compared to GpA and in the UpG/CpA and GpG/CpC pairs (Additional file 2: Figure S1B).

### Fitting the mutational model to observed dinucleotide frequencies

Observations of differing dinucleotide representations in DNA and mRNA sequences and the asymmetries between complementary pairs in mRNA justified the development of separate mutational models for DNA and mRNA sequences. To investigate which context dependent substitutions could account best for the pattern of dinucleotide under- and over-representation in each sequence dataset, we developed a Markov process parameter estimation method. This evaluated every possible substitution with each upstream and downstream neighbouring base and an associated mutation rate that maximized the fit between modelled and observed dinucleotide frequencies. The degree of fit was quantified by calculation of root mean square (RMS) distances between modelled frequencies for sequences of different G+C contents and those of a sample of actual sequences.

For RNA, there were 96 possible context-dependent mutations, while the symmetry of DNA allowed 48 (*eg.* C→T,G is formally equivalent to C,G→A; see Methods). For both datasets separate optimization of transition / transversion ratios (*κ*) represented an additional parameter applied to mutations that was incorporated into the modelling process. The parameters that produced the lowest RMS distance for all 16 dinucleotides was selected. Since the model was fitted to sequences with a range of G+C contents, RMS distances are additionally influenced by how well the model reproduces the marked G+C dependence in the frequencies of certain dinucleotides (such as CpG and UpA).

Having identified the context-dependent mutation, its rate and *κ* that best fitted the observational data, the mutation (although not its rate) was fixed and the analysis repeated to find a second context-dependent mutation that in combination with the first and a re-optimised value of *κ*, created the greatest further reduction in RMS distances. This procedure was repeated with further context-dependent mutations until there was no further reduction in RMS distance. Fitting two parameters to human DNA and mRNA sequences led to a better match between the model predictions for UpA and CpG dinucleotide frequencies (red and blue lines respectively) across a range of G+C compositions than achieved using one parameter. Using four parameters provided a better match than two and indeed modelled CpG and UpA frequencies very closely matched the quadratic line of best fit through the observational data (black lines; Figure 2).

Matches between model predictions and observed frequency data extended to other dinucleotides in human DNA sequences and in most cases also successfully reproduced relationships between dinucleotide frequencies and G+C content (Additional file 2: Figure S1A). Similarly close fits between modelled and observational data for the 16 dinucleotides were observed for human mRNA sequences (Additional file 2: Figure S1B). Model predictions additionally reproduced the observed differences in frequencies of self-complementary dinucleotides (such as CpA and UpG), represented as red/yellow/white and dark blue/light blue/white filled symbols.

Modelling was extended to DNA and RNA datasets for the other organisms (data from a fish and an insect are shown in Additional file 2: Figure S1C, S1D, S1E and S1F). Similarly close fits between modelled and observed frequencies were observed at each using up to 4 parameters. The main difference from human sequences was the much more restricted range of G+C contents of both DNA and mRNA sequences in each that made fitting the data to G+C compositional trends less relevant.

### Quantifying model error

To quantify how well our model fitted the observational data, RMS distances between the observed dinucleotide frequencies and those predicted by the model were calculated for all 16 dinucleotides. These were then compared with the corresponding minimum RMS distances between the observed dinucleotide frequencies and a separate quadratic model of each dinucleotide. These quadratic models yield the best possible fit to the data and lowest possible RMS value. Any other model will have higher RMS values and the amount by which its RMS values are above the quadratic models’ RMS values shows the error in the model. We refer to this as the baseline corrected model error and this is used in the presentation of the RMS results. As an example, the best fit model data for mammalian DNA using 3 mutation rates had a RMS distance for all 16 dinucleotides of 0.0378 while quadratic best fit data showed a RMS distance of 0.0275. The baseline-corrected model error was therefore 0.0103 (0.0378 – 0.0275). The calculation of baseline corrected model errors therefore excludes measurement errors associated with dinucleotide frequency measurements of often relative short nucleotide sequences.

The effect of sequence length on RMS calculations can be visualised by comparison of the degree of scatter of dinucleotide frequencies of human mRNA sequences (mean length of approximately 2463 bases) with that of the much longer DNA sequences (50,000 bps; Figures 1A, 1D, 2A, 2B). To more formally demonstrate the relationship between RMS scores and sequence lengths, human DNA sequences of lengths ranging from 400,000 to 500 bps were generated and model error estimated for each dataset using separate modelling to minimum values (Additional file 3: Figure S2 in Supplementary Data). An empirical relationship between sequence length and RMS distance can be represented as:

$$\mathrm{RMS}\;\text{distance}=\left(2.2/{\text{length}}^{0.42}\right)+0.0095$$

The intercept with the y-axis of 0.0095 therefore represents model error for DNA fragments of infinite length (*ie*. not attributable to sampling error). For DNA fragments of 50,000 bps in length, sampling error can be estimated to contribute 0.0329 to RMS distances, while for human mRNA sequences, sampling error was three times higher at 0.0994. These values are close to RMS distances calculated from lines of best fit to the data (0.0275 and 0.0972 respectively). This close match for human sequences was reproduced in corresponding datasets for other organisms (Additional file 4: Table S2).

### Effectiveness of context-dependent mutational rate modelling

**Figure 3:**
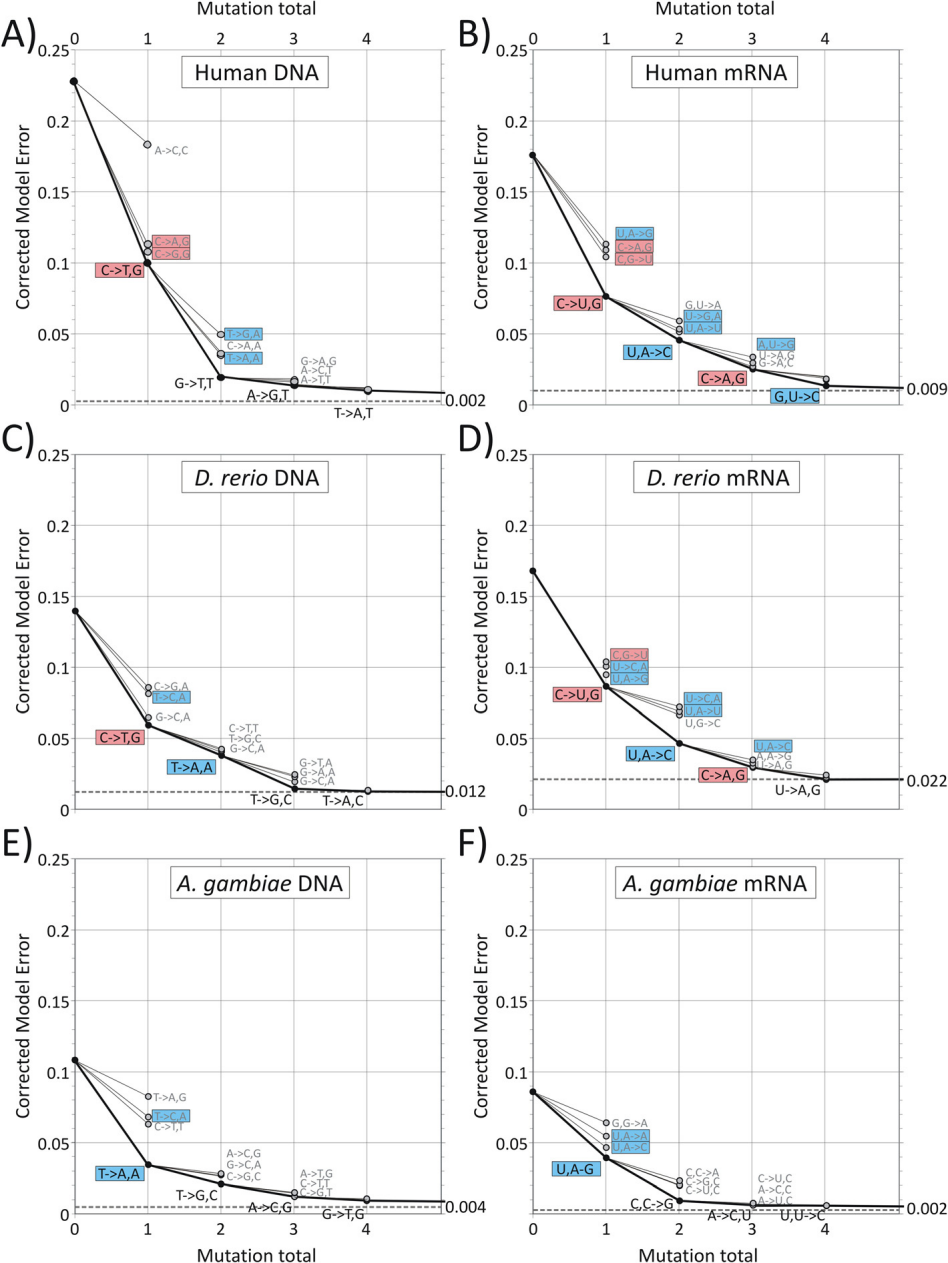
**Model errors (y-axis) for mutational models with between 1 to 4 context-dependent mutational biases for vertebrate (human, *D. rerio *) and non-vertebrate ( *A. gambiae *) DNA and mRNA sequences.** Minimum RMS distances for up to 16 additional mutational biases are shown as a dotted line. Model error reductions for alternative mutational biases are shown as unfilled circles. Mutations that remove CpG and T/UpA are shown in pink and blue inset boxes respectively. All model error values have been baseline corrected by subtraction of RMS scores of quadratic lines of best fit through observational data.

Model errors for the first four most influential mutations and the minimum value were calculated for human, fish and insect datasets (Figure 3). All values were baseline corrected by subtraction of RMS scores of quadratic lines of best fit through observational data (uncorrected RMS scores are shown in Additional file 5: Figure S3). For non-cytoplasmically expressed human DNA sequences, corrected model errors fell from an initial value of 0.228 (no context-dependent mutations) to 0.0024 (minimum value achieved with 9 parameters; Figure 3A). The two most influential context-dependent mutations were C→T,G (model error reduction to 0.100) followed by G→T,T (0.019) with minimal proportionate reductions using further mutations. These model error reductions correspond to the successively better fits between modelled and observed frequencies for human DNA and mRNA for UpA and CpG dinucleotides displayed in Figure 2. Both the mutations and their mutation rates were highly reproducible on replicate sampling of DNA sequences (Additional file 6: Table S3). Similarly, for organisms such as the three mammalian species in which we suspect similar mutational biases and selection pressures may exist, the first three context-dependent mutations were, with one exception, identical while model error reductions, values of *κ* and mutation rates were highly similar (Table 1).

**Table 1:** Comparison of the first three context-dependent mutations and mutation rates in three mammalian species

**Seq.**	**Species**	***κ***	**Corrected model error**	**1**^**st**^	**Rate**	**2**^**nd**^	**Rate**	**3**^**rd**^	**Rate**
DNA	*H. sapiens*	3.1	0.0135	C→T,G	12.06	G→T,T	6.16	A→G,T	1.42
	*P. troglodytes*	4.6	0.0134	C→T,G	11.29	G→T,T	6.01	A→G,T	1.30
	*M. musculus*	4.6	0.0117	C→T,G	14.33	G→T,T	4.30	C→G,A	2.20
RNA	*H. sapiens*	1.7	0.0249	C→U,G	10.26	U,A→C	9.53	C→A,G	9.40
	*P. troglodytes*	1.6	0.0265	C→U,G	10.17	U,A→C	11.93	C→A,G	10.00
	*M. musculus*	1.6	0.0183	C→U,G	10.84	U,A→C	9.48	C→A,G	10.73

A further insight into the robustness of these predictions was obtained by plotting out baseline corrected model error for 2^nd^, 3^rd^ and 4^th^ ranked alternative context-dependent mutations. For human DNA, C→T,G and G→T,T led to a substantially greater reduction than alternatives despite their frequent similarities in their effects on sequence composition. For example, 2^nd^ and 3^rd^ alternatives to C→T,G also eliminated CpG residues from sequences (shaded pink boxes; Figure 3A). The same findings were obtained on analysis of DNA sequences of other mammalian genomes (*M. musculus* and *P. troglodytes*; data not shown)*.*

### Context-dependent mutations and single nucleotide polymorphism (SNP) frequencies

**Figure 4:**
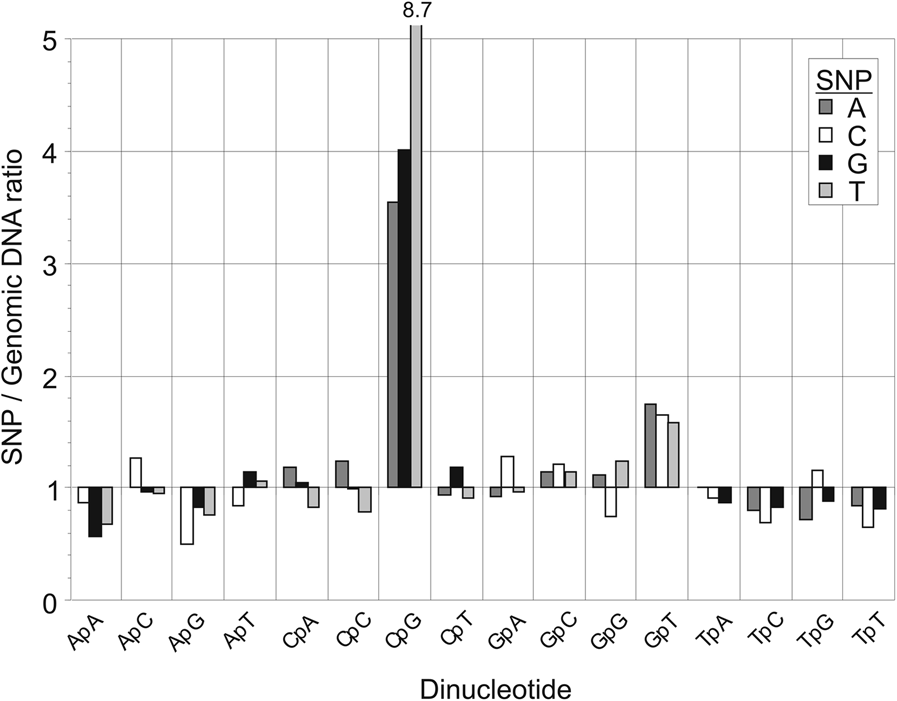
**Frequencies of SNPs (y-axis) occurring in each dinucleotide context (x-axis) compiled from approximately 45 million SNPs in human genomic DNA sequences.** These were categorised by mutation type and by the base downstream of the SNP (3′ dinucleotide context). Frequencies of SNPs were normalised to those predicted from dinucleotide frequencies and transition and transversion rates measured in the whole SNP dataset.

The consistent prediction in mammalian datasets of the G→T,T mutation, ranked second, was unexpected and did not correspond to any characterized mutational bias in mammalian genomes. To investigate whether there was greater mutability of the GpT dinucleotide in human DNA sequences, we compiled a large dataset of approximately 45 million SNPs compiled from the NCBI dbSNP database and compiled frequencies of each possible mutation (*ie.* A↔C, A↔G, …..G↔T) subdivided into groups according to the base downstream of the SNP (3′ dinucleotide context). These frequencies were normalized by frequencies of each dinucleotide and of each mutation in the human SNP dataset to calculate the influence of dinucleotide context on mutation frequencies. As expected, SNP mutations showed a strong preference for the first ranked C→T,G mutation (8.6× expected frequency) predicted from modeling and additionally the C→A,G and C→G,G alternative mutations (3.5× and 4.0× respectively; Figure 4). Consistent with the second ranked mutation detected on modelling, consistently elevated mutational frequencies were observed in the GpT dinucleotide (1.6× – 1.8×) although in this case there was a less clear bias among the three possible mutations for the G↔T transversion predicted in the model. The instability of GpT identified by SNP analysis supports the prediction of an elevated G→T,T mutation rate identified by modelling and by the under-representation of GpT in human (Additional file 2: Figure S1A; ≈80% of expected value) and in other mammalian sequence datasets (data not shown).

### Mutational biases in mRNA sequences

Differences in dinucleotide composition between human non-cytoplasmically expressed genomic DNA sequences and mRNA sequences were reflected in different best-fit mutational models between DNA and RNA sequences (Figure 3). The composition of mRNA sequences is influenced by mutational pressures operating on the underlying DNA sequences, as well as possible mutational biases introduced by RNA polymerase II and by selection pressures in the cytoplasm. While the most influential mutation was C→U,G, along with alternatives that also removed CpG dinucleotides, the second (and alternatives) all removed UpA dinucleotides, a mutational or selection pressure absent in mammalian DNA sequences. mRNA showed further mutations that removed CpG and UpA dinucleotides, consistent with a greater, possibly cytoplasmically-driven selection pressure to remove these two dinucleotides (see Discussion). Evidence for greater complexity of the mutation and/or selection pressures operating on mRNA sequences was provided by the greater number of mutations needed to reduce model error and larger minimum value from the best fitted model.

For DNA sequences of other species, mutations removing CpG dinucleotides were found among those with methylated genomes (*D. rerio;* Figure 3C along with the sea squirt*, C. intestinalis*) as expected but were entirely absent among *A. gambiae* sequences (Figure 3E) and other organisms with non- or weakly-methylated genomes (*D. melanogaster, C. elegans*; data not shown). mRNA sequences from all species showed a predominance of mutations that removed UpA, consistent with widespread cytoplasmically driven selection against this dinucleotide.

**Figure 5:**
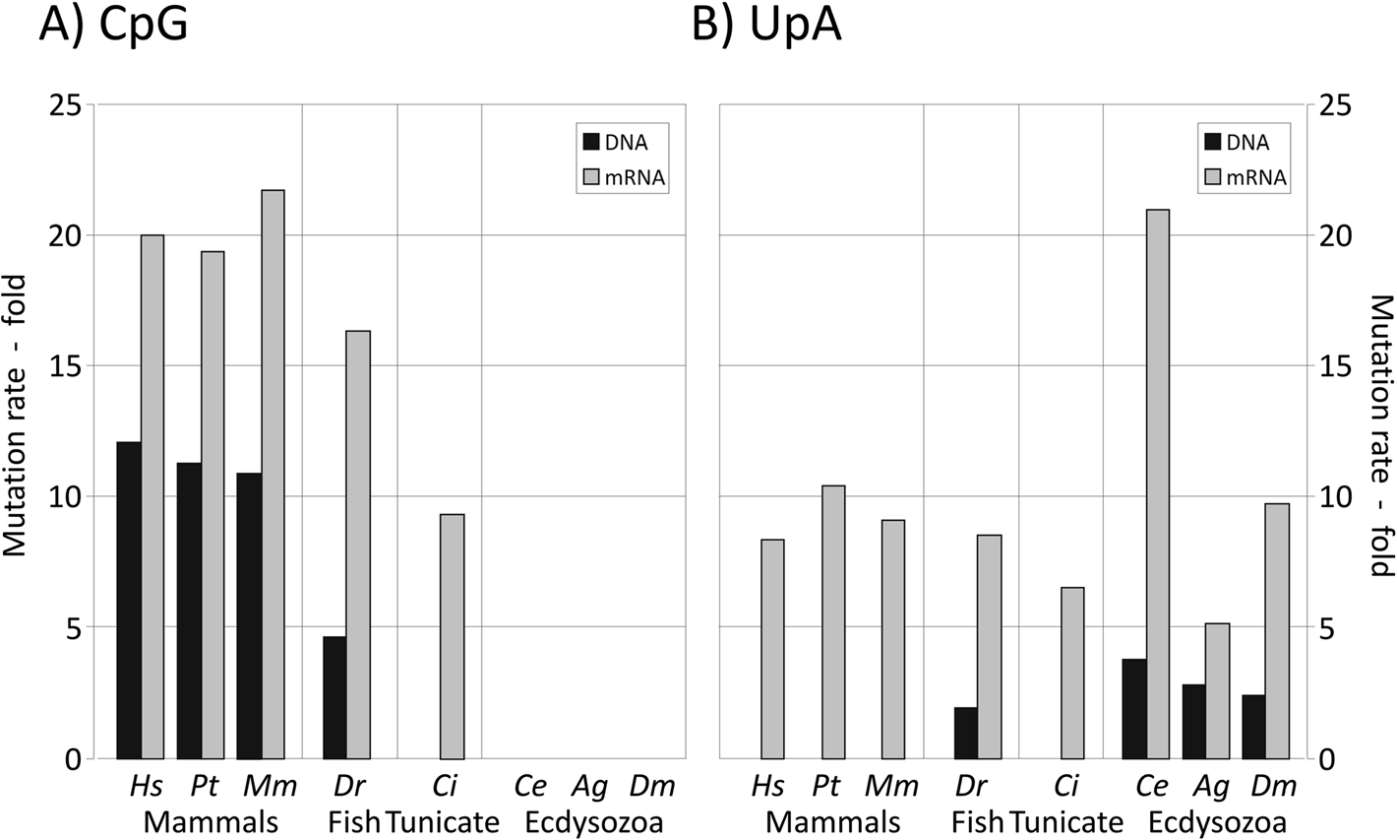
**Mutation rates for sequence changes that remove CpG and UpA dinucleotides in different eukaryotes. (A)** Rates for mutations removing CpG dinucleotides among the first three parameters for genomic DNA and mRNA sequences of different eukaryotes **(B)** Mutations removing UpA dinucleotides. Zero values indicate that mutational biases were not detected. Abbreviations: HS: H. sapiens; Pt: P. troglodytes; Mm: Mus musculus; Dr: D. rerio; Ci: C. intestinalis; Ce: C. elegans; Ag: A. gambiae; Dm: D. melanogaster.

To compare mutational and/or selection pressures operating against CpG and UpA dinucleotides in different organisms, modelled mutation rates (−fold excess over default values) were calculated for the most influential mutations that remove these in each species (Figure 5). This analysis confirmed the absence of mutational or selection pressures against CpG dinucleotides in DNA or mRNA sequences in any of the organisms with non-methylated genomes (ecdysozoa). In contrast, selection against UpA dinucleotides was universal in mRNA sequences of all organisms examined and occurred at an optimized rate that was invariably several fold higher than observed in corresponding DNA sequences. Mutations removing TpA was indeed absent in all mammalian datasets and in *C. intestinalis* among the first four parameters that were most influential in reducing model error.

Unexpectedly, greater mutational rates in mRNA sequences compared to non-cytoplasmically expressed DNA sequences were also observed for CpG dinucleotides, where modelled rates were consistently higher (despite the existence of the methylation-induced mutational pathway operating on genomic DNA sequences of vertebrates). The existence of an additional selection pressure imposed on cytoplasmically expressed sequences was consistent with the existent of two mutations rather than one (C→U,G [1st parameter] and C→A,G [3^rd^ parameter]) in human mRNA sequences and in other mammalian mRNA datasets (*P. troglodytes, M. musculus;* Table 1; Figure 3B).

### Modelling mutational and selection biases in mammalian RNA viruses

**Figure 6:**
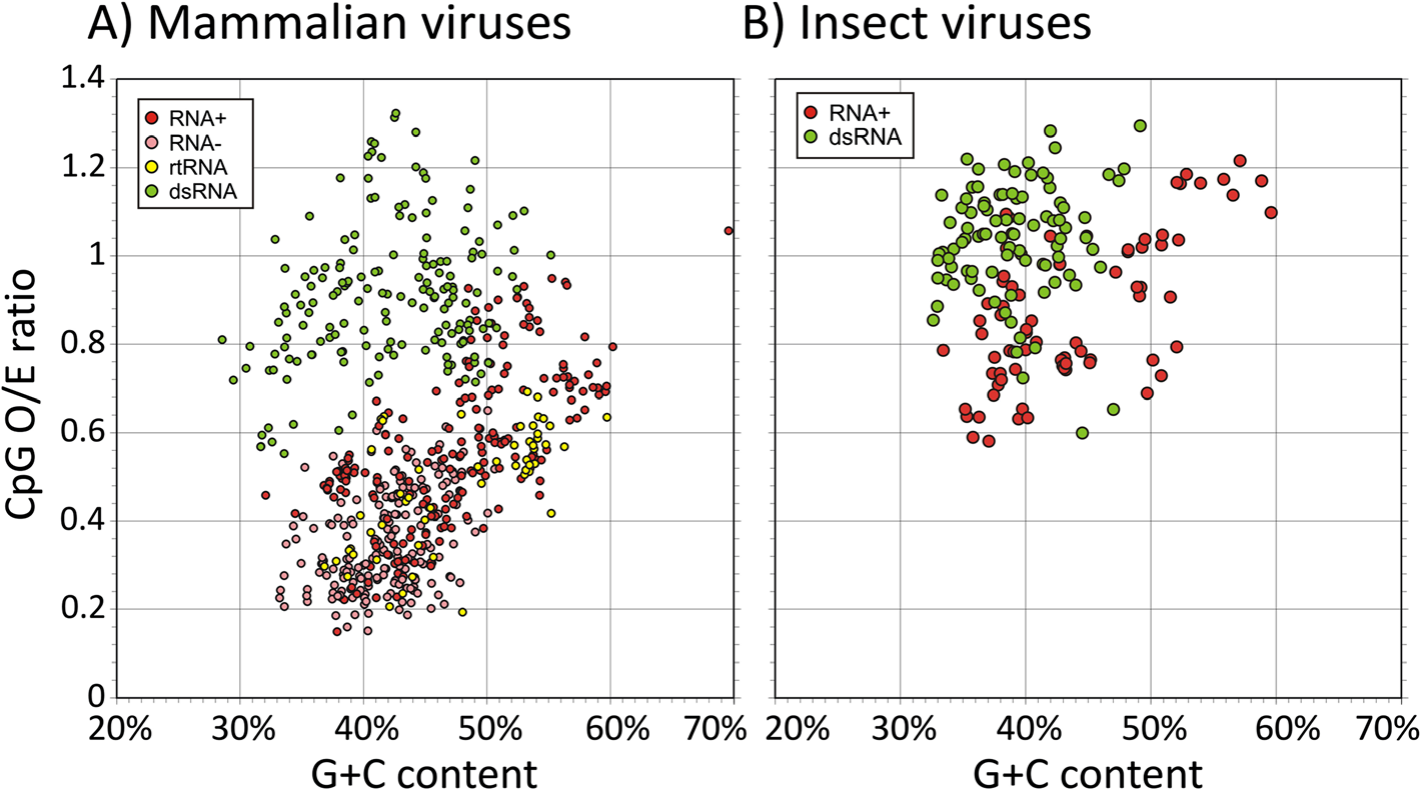
**CpG dinucleotide frequencies among different classes of RNA and small DNA viruses infecting mammals and insects.** Viruses were divided into groups based on the genomic composition: RNA+: positive strand RNA viruses; RNA-: negative strand RNA viruses; rtRNA: reverse transcribing viruses (Retroviridae, Hepadnaviridae and Caulimnoviridae); dsRNA: double-stranded RNA viruses; ssDNA: single stranded DNA viruses as indicated in the inset box.

RNA viruses replicate in the cytoplasm of a wide range of eukaryotes and are potentially susceptible to the same selection pressures observed in host mRNA sequences. To investigate this, dinucleotide compositions in complete genome sequences from a wide range of RNA and small DNA viruses infecting mammals and insects were calculated. Consistent with previous analyses [10], most classes of RNA virus and small DNA viruses showed evidence of marked CpG suppression (Figure 6) and a wide range of under-and over-representation of other dinucleotides (Additional file 7: Figure S4A, S4B). No suppression of CpG was apparent among insect viruses. RNA viruses were subdivided into groups based on the configuration of their genomic RNA (based on the Baltimore classification) and potential exposure to the cytoplasm. RNA viruses with single stranded genomes (positive or negative sense) and reverse transcribing viruses (*eg.* retroviruses) showed similar degrees of CpG suppression that was related to their G+C composition, while no comparable suppression was observed in dsRNA viruses (Figure 6; green filled circles *p* < 10^-10^; Additional file 1: Table S1). These observations provided tentative evidence that RNA viruses that expose their genomic RNA sequences to the cytoplasm are subject to similar selection against CpG as was evident in mRNA sequences. Insect viruses of any configuration showed no CpG under-representation.

**Figure 7:**
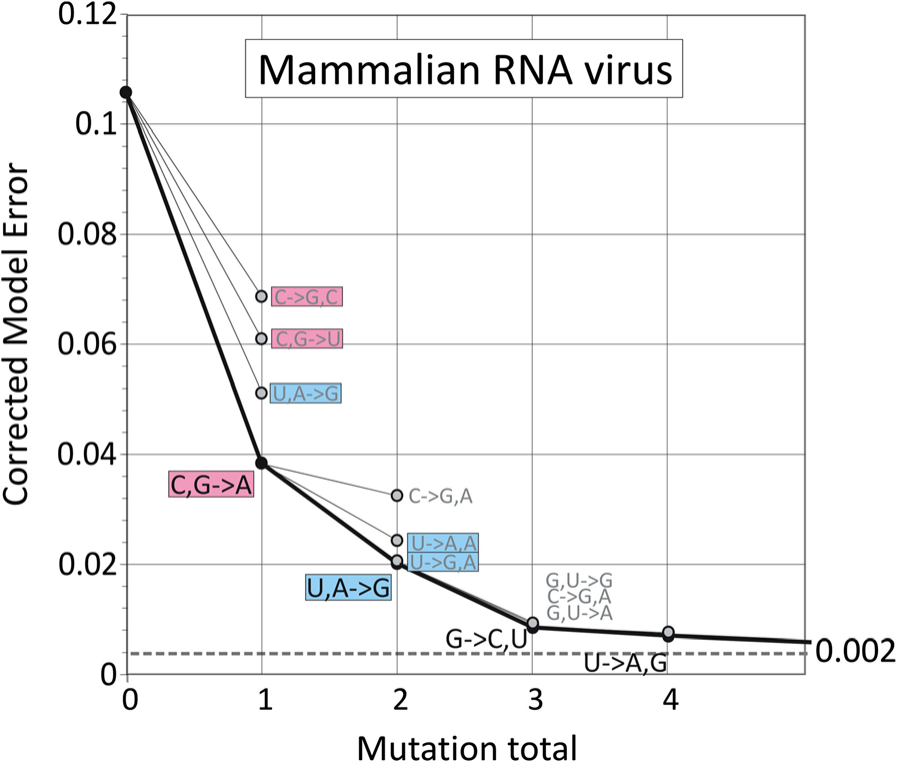
**Baseline corrected model errors (y-axis) for mammalian RNA viruses using mutational models with 1 to 4 context-dependent mutational biases (labeled under graph line) and minimum values using up to 8 additional mutational biases (dotted line).** Model errors for alternative mutational biases are shown as unfilled circles. Mutations that remove CpG and UpA are shown in pink and blue boxes respectively.

To investigate whether the suppression of CpG in RNA viruses was a response to similar mutational and selection pressures observed in their hosts’ mRNA sequences, 420 animal positive- and negative- sense viruses were analyzed using the 96 parameter mutational model (Figure 7; uncorrected RMS scores are shown in Additional file 8: Figure S5). As observed among mRNA sequences of their hosts, the main context-dependent mutations that reduced model error were those that eliminated CpG and UpA dinucleotides, prominently represented among both the first choice and alternative mutations.

## Discussion

### Modeling mutational processes

This study investigated several unresolved issues in previous analyses of dinucleotides and the context-sensitive mutational and selection biases. Specifically, are simple processes such as the elevated C→T transition frequency upstream of G residues arising from methylation necessary and sufficient to account for the spectrum of skewed dinucleotide frequencies observed in mammalian genomic DNA sequences? A previous investigations of dinucleotide composition of genomic sequences in a range of eukaryotic phyla showing different degrees of methylation and CpG under-representation demonstrated (despite previous reports to the contrary; *eg.*[18]) that the observed CpA/TpG over-representation arose in direct proportion to the loss of CpG dinucleotides [13]. Using a modeling method on which the current study was based, Duret and Galtier [14] further showed that assigning an elevated C→T,G rate upstream of G residues reproduced the G+C relationship with CpG under-representation in human genomic DNA and, rather counter-intuitively, additionally reproduced the G+C-dependent depletion of TpA dinucleotides also observed in human DNA sequences [14]. Despite the title of that study however, the actual depletion of UpA is proportionately greater in genomic DNA than could be modelled and the further effect of this primary mutational bias on other dinucleotide representations was not analyzed. A further problem with this hypothesis is that UpA deficiencies are equally pronounced among organisms that lack methylation of genomic DNA and show no suppression of CpG frequencies (*eg. A. gambiae -* Additional file 2*:* Figure S1E).

In the current study, we have substantially expanded the modelling process to allow multiple mutational biases and rates and used model error calculations to allow each to be systematically optimized rather than empirically assigned. The method proved robust, with minimal variability in predicted mutations and mutational rates of human DNA when different random selected samples were analyzed (Additional file 6: Table S3) or on comparison of mammalian genomic DNA and RNA datasets where selection pressures are expected to be similar between species (Table 1; Figure 5). We do acknowledge, however, that finding the simplest combination of context-dependent mutations and associated mutational rates that fits the observational data is not necessarily the actual underlying biological process. However the mutational models we have discovered are compelling in their simplicity, efficiently account for observational data with a minimum of parameters and predict context-dependent mutations and rates that are both biologically plausible and consistent with results and inferences made from different approaches [19-22]. This applies particularly to mammalian DNA datasets where the use of just two mutations reduced baseline corrected model error to close to zero. Furthermore, the optimized mutational rate for the C→T,G transition was comparable to estimates based on different methods. For example a 12-fold higher rate compared to other transitions was reported using a simple equilibrium model [22]. More recent maximum likelihood approaches that incorporate the C→T,G transition rate in human genomic DNA as a separate parameter to standard substitution models for likelihood-optimization, arrive at mutational rates ranging from 8.5 (TF model; [19]) to 9.2 [21], similar to the modelled 11.0× – 12.1× rates we derived for mammalian DNA (Table 1).

One unanticipated finding that supports the validity of the modelling method was the reconstruction of the relationship between the under-representation of CpG and TpA (and other dinucleotides) with G+C content. Quadratic lines of best fit through observational data superimposed almost exactly on model predictions using 4 or fewer parameters Figure 2 and Additional file 2: Figure S1). This provides a simpler explanation than hypotheses that propose different susceptibilities of high and low G+C content DNA to methylation and deamination or different selection pressures operating on CpG islands that contain higher proportion of coding sequences [23]. For example, one widely discussed model argues that genomic DNA with a low G+C content is more susceptible to methylation-induced mutations that eliminate CpG dinucleotides [8,9]. The effect of replacing C with T further reduces G+C composition in these regions encouraging further methylation and elimination of CpG dinucleotides. The theory provides a compelling explanation for the existence of alternating regions of low C+G content and heavily methylated DNA interspersed with CpG-rich islands (particularly in warm-blooded animals where elevated temperatures potentially contributes to the accessibility of low G+C DNA to methylation). However, we have found that precisely the same relationship emerges from a model in which G+C content had no influence on methylation rate.

Compared to this simple, single parameter modelling previously reported of human DNA [14], at least one further mutation and better optimized C→T,G mutation rate and *κ* (transition / transversion) ratio was required to reproduce the steeper positive (CpG) and negative (TpA) gradients between dinucleotide representation and G+C content. In the case of TpA, the use of two parameters additionally reproduced the degree of under-representation of TpA observed in genomic sequences that was not effectively modelled in the original study.

Previous investigation and modelling of mutations that create dinucleotide frequency biases have typically concentrated specifically on CpG and its under-representation in mammalian genomes. There is therefore a dearth of published information to corroborate predictions for other dinucleotides and among other organisms without genomic methylation. There is for example little information on the potential existence of the highly influential G→T,T mutation identified in mammalian DNA sequences, C→T,T in *D. rerio* and G→A,A among ecdysozoa. The GpT dinucleotide is depleted in mammalian DNA sequences as well as in eubacterial and mitochondrial genomes [24], and consistent with the greater than expected frequency of SNPs involving this dinucleotide in a large scale analysis of human SNP data (mean 1.7-fold; Figure 4). This mutational bias is indeed visible although uncommented on in previous SNP analyses of human and mouse sequences [25,26]. Together these findings are consistent with a greater mutability of this dinucleotide.

### Differential selection on expressed mRNA sequences

In contrast to previous studies, investigation of mutational and/or selection biases was based on genomic sequences separated into expressed directly as mRNA sequences and DNA sequences that are non-transcribed. This differentiation was particularly relevant for organisms with high proportions of coding and other expressed sequences in their genomes. This differentiation revealed several differences both in their dinucleotide frequency biases and in the optimised models for their underlying mutational and selection biases.

The first observation was that dinucleotide frequency biases were often distinct between genomic DNA and mRNA sequences, even though the latter sequences necessarily incorporate mutational processes operating on genomic DNA. The proportionately greater under-representation of UpA dinucleotides for a given G+C content observed in mRNA sequences has been previously described [7], although this phenomenon extends to several other dinucleotides which show even greater compositional differences (such as GpA and CpA in human mRNA; Additional file 2: Figure S1B). Further evidence that different selection may be operating on mRNA sequences was indicated by frequent asymmetries in complementary dinucleotides, such as CpA and UpG that could not have originated through mutational biases occurring on genomic DNA (where they are effectively symmetrical). Mutational models developed for mRNA sequences showed several further differences from those optimised for genomic DNA sequences of the same organism. Most prominent was the evidence in all species examined for strong selection against the UpA dinucleotide, ranked first or second in order of influence. In contrast, selection against UpT was either absent (mammalian species, *C. intestinalis*) or substantially weaker in genomic DNA sequences (Table 1, Figure 5). Best fitting mutations that removed UpA residues were usually transitions (*eg.* U→C,A) but showed no evidence of context dependence that would be expected for a mutational bias. Similarly, among the species investigated, the 96+1 parameter (asymmetric) model generated similar numbers of upstream and downstream-base conditioned mutations, such as U,A→C in mammalian mRNA and *D. rerio* and U,A→G in *A. gambiae* (Table 1, Figure 3). This contrasted with the strict dependence of methylation-induced transitions on a downstream G residue in DNA sequences.

Selection against UpA dinucleotides in cytoplasmically-expressed sequences might be expected given the role of the UpA dinucleotides as a recognition motif for RNAseL and other RNA degrading enzymes [6,7,27]. For example, human mRNA sequences expressed in the cytoplasm of CHO (hamster) cells showed greater degradation rates in proportion to frequencies of UpA residues in the cytoplasm [6]. Although there is little information on degradation pathways of mRNA sequences in invertebrates, the observation that UpA is consistently under-represented throughout eukaryotic phyla provides some evidence for the existence of comparable regulatory mechanisms [7]. As suggested many years ago, the suppression of UpA dinucleotides among RNA viruses infecting mammalian, plant and insect cells (Figure 6; [10,11]) may therefore represent their specific adaptation to evade RNA degradation during their replication cycle. In the current study, further evidence for specific selection against UpA dinucleotides was provided by the mutational model for positive- and negative-strand animal viruses in which mutations removing UpA residues were ranked second behind those removing CpG (Figure 7).

### Selection against CpG dinucleotides in expressed RNA sequences

Mutations eliminating CpG residues were also highly influential in reducing model error for mRNA sequences and ranked 1^st^ or 2^nd^ in species with methylated genomes (Figure 3). Although these arise (at least in part) from mutational biases in the underlying genomic sequence, modelled mutational rates were invariably higher in mRNA sequences (Figure 5). Furthermore, in *C. intestinalis,* CpG depletion was best modelled by mutations that were dependent on the upstream base (*eg.* C,G→A; Figure 3 and data not shown). These observations provide evidence that additional, likely selective rather than mutational pressures against CpG dinucleotides are exerted on RNA sequences expressed in the cytoplasm. The existence of this selection pressure operating independently of DNA methylation induced mutation is supported by our finding of mutational biases against CpG dinucleotides among RNA viruses (Figure 7) in which conventional deamination and mutation as a consequence of methylation cannot occur. This selection process may underlie the prominent under-representation of CpG dinucleotides in many classes of RNA virus [10,11] infecting mammals and plants to extents comparable to those observed in their hosts’ mRNA sequences (Figure 2, Additional file 7: Figure S4A, S4B; [28]). Prominent exceptions to CpG under-representation are viruses with dsRNA genomes (Figure 6) and many of the helical-classed plant viruses (data not shown). In these, however, RNA genomic sequences remain packaged within virions throughout their replication cycle and they therefore may not be subject to the same selection pressures operating on exposed RNA.

It could be argued that host cell defences against viral infections that mutate their RNA genomes may account for the various under- and over-representations of specific dinucleotides. Of these, members of the APOBEC family deaminate cytosines in single-stranded DNA and RNA potentially in specific sequence contexts [29] although those identified (C,C->U and U,C->U) would not create the dinucleotide biases in RNA viruses and of course the action of APOBEC is specific to retroviral genomes, not the RNA viruses modelled in the current study. A different RNA editing enzyme that is interferon-induced and known to be active against RNA viruses is adenosine deaminase acting on RNA 1 (ADAR1). However, its mutagenic effect is not known to be dependent on dinucleotide context [30] and therefore similarly cannot create the frequency biases observed.

Although the nature of the selection against CpG dinucleotides remains poorly understood and has not been investigated functionally, there are a number of tantalizing clues towards the existence of mechanisms coupled to innate immunity that recognize RNA with CpG motifs [28,31,32]. There may be, for example, RNA-degrading enzymes that recognise CpG motifs, analogous to UpA targeting by RNAseL and other RNA degrading enzymes that influence mRNA half-lives in the cytoplasm (see above). Alternatively, CpG dinucleotides in viral RNA may be selected against as they may serve as targets for currently uncharacterized pathogen recognition receptors couple to interferon or other cell defence pathways [12]. The induction of interferon-β in macrophages exposed to synthetic RNA oligonucleotides containing CpG residues [33] may be an example of this process, functionally and perhaps evolutionarily related to Toll-like receptor 9 that recognizes non-methylated CpG dinucleotides in DNA sequences.

Further evidence that the presence of CpG dinucleotides in viral sequences either activate or are targets of cell defence mechanisms is provided by the observation that polioviruses with artificially elevated CpG frequencies in their genomic RNA were markedly attenuated and replicated to titres several orders of magnitude lower than wild type virus in *in vitro* cell culture [34-36]. Intriguingly, cellular genes coding for proteins induced as part of the innate response to infection, such as type 1 interferons, show substantially greater depletion of CpG dinucleotides than other genes of similar G+C composition [31], suggesting that this adaptation is required for effective gene expression in a hostile cytoplasmic environment. Mammals (and potentially other vertebrates) and plants with their methylated genomes and associated depletion of CpG may therefore have independently co-opted this dinucleotide as a marker of self/non-self recognition. This potentially explains the selection against CpG in viruses infecting members of these eukaryotic phyla [28]. The existence of such recognition systems may in turn have placed additional selection pressures on host expressed mRNA sequences to evade these viral countermeasures.

## Conclusions

The findings in the current study provide the first comprehensive analysis of context-dependent mutational biases and selection pressures in organisms with both methylated and non-methylated genomes. The finding of pressures operating on genomic DNA in addition to the previously described C→T,G mutation in mammals, a set of quite different biases in non-methylated genomes and additional selection pressure operating on sequences expressed as mRNAs in all organisms provides a series of predictions that can be directly analyzed in biological studies. The evidence obtained for selection pressures against UpA and CpG dinucleotides in mRNA sequences of methylated organisms provides a coherent explanation of their under-representation in cytoplasmically replicating RNA viruses which has eluded previous analyses [10,11]. It provides exciting new insights into the process of self / non-self recognition that underlies host innate immunity to viral pathogens.

## Methods

### Sequences and dinucleotide frequency calculation

DNA sequences from human (*Homo sapiens*), other mammals (chimpanzee [*P. troglodytes*], mouse [*M. musculus*]), another vertebrate (zebra fish [*D. rerio*]) and other animals (sea squirt [*C. intestinalis*], fruit fly [*D. melanogaster*], mosquito [*A. gambiae*] and nematode [*C. elegans*]) were the subject of the investigation. Genome sequences were obtained from UCSC for the following genome versions: *H. sapiens -* hg19; *P. troglodytes* - panTro3; *M. musculus* - mm9; *D. rerio -* danRer7; *C. intestinalis -* ci2; *C. elegans* - ce10; *A. gambiae* - anoGam1; *D. melanogaster*, dm3.

Exon coordinates were extracted from UCSC using the table browser function using the following tables: hg19: knownGenes; panTro3: refGene; mm9: knownGenes; danRer7: refGene; ci2: refGene; ce10: refGene; anoGam1: refGene; dm3: refGene. Sequences corresponding to exon coordinates were removed and the remaining non-cytoplasmically expressed DNA genomic sequences were divided into 50,000 bp lengths for analysis.

From each species, non-redundant mRNA sequences were downloaded from the http://www.ncbi.nlm.nih.gov/gene database, with sequences shorter than 2500 bases excluded. Complete genome sequences from available positive and negative stranded RNA viruses infecting mammals were obtained from GenBank (Additional file 9: Table S4). The analysis used non-redundant sequences curated in the RefSeq project comprising prototype or reference sequences from each virus family, and species.

SNPs in human DNA and their immediate 5′ and 3′ bases were obtained from the NCBI dbSNP database (ftp://ftp.ncbi.nih.gov/snp/organisms/human_9606/rs_fasta/) on 18/01/12. The bases immediately adjacent to the 44,415,612 SNPs were extracted by parsing FASTA files, ignoring insertion/deletion polymorphisms.

Mono- and dinucleotide frequencies and ratios of observed dinucleotide frequencies to those expected from mononucleotide composition (G+C content in the case of DNA sequences) were calculated using the program Composition Scan in the SSE package [37].

### Modelling substitution rates in different dinucleotide contexts

We developed a systematic model to determine optimal mutation rates in each dinucleotide context that best correlate with DNA and RNA composition of eukaryotic and viral sequences. These rates can viewed as variations from a default rate transformation matrix, *Q*:

$$\begin{array}{l} \;C\;G\;U\;A\\ Q\;=\;\begin{array}{c} C\\ G\\ U\\ A \end{array}\;\left[\begin{array}{cccc} • & \theta & \kappa \left(1-\theta \right) & 1-\theta \\ \theta & • & 1-\theta & \kappa \left(1-\theta \right)\\ \kappa \theta & \theta & • & 1-\theta \\ \theta & \kappa \theta & 1-\theta & • \end{array}\right] \end{array}$$

where *Q*_*YW*_ for distinct *Y* and *W* is the default rate of transformation from nucleotide *Y* to nucleotide *W*. *κ* is the transition to transversion ratio and, for the default transformation rates given by the matrix *Q, θ* is the equilibrium proportion of G+C mononucleotides. It is assumed that these rates can be influenced independently by the two neighbouring nucleotides to *Y* as follows. For a given trinucleotide *XYZ* the mutation rate from *Y* to a different nucleotide *W* is given by:

$$r\left(X,Y\to W,Z\right)=f\left(X,Y\to W\right)\;Q_{\mathrm{YW}}\;f\left(Y\to W,Z\right)$$

where *f(X, Y→W)* is the factor giving the change of the mutation rate of *Y→W* from its default value when the upstream nucleotide is *X*, and *f(Y→W, Z)* is the factor when the downstream nucleotide is Z, and both factors contribute independently. For example if *f(X, Y→W)* and *f(Y→W, Z)* were changed from their default values of 1 to values 0.7 and 2, then the mutation rate *r(X, Y→W, Z)* would increase by a factor of 1.4 from its default value of *Q*_*YW.*_ This model generalizes Duret and Galtier’s model [14], in which *κ* = 2.1, *f*(C→T, G) = 27.6/2.1 and the rest of factors all equal to 1.0.

For each of the 4 nucleotides, *Y,* there are 3 possible transitions to a different nucleotide, *W* giving 12 possible transitions *Y→W*. Since there are 4 possible upstream nucleotides *X*, there are 48 factors of the form *f(X,Y→W)* and since there are 4 downstream nucleotides *Z* there are 48 factors of the form *f(Y→W, Z)*, giving a total of 96 factors in the model. (Note that this is half the number of factors that would be needed in a model that had a factor for each of the 12 possible transitions and each of the 16 combination of upstream and downstream nucleotides.) In RNA all the 96 factors in the model are independent. However in DNA there is strand symmetry which leads to equal rates of mutation in complementary DNA strands. Consequently if *X’*, *Y’* and *W’* are the complementary nucleotides to *X*, *Y* and *W* respectively, then *f(X, Y→W)* = *f(Y’→W’, X’)*. Hence for DNA there are only 48 independent factors.

For any specified set of mutational rates *r(X, Y→W, Z)* we can simulate the mutational process starting from some arbitrary compositions until an equilibrium is reached. The method used is as follows.

Let *d*_*ij*_*(u)* be the proportion at time *u* of all the dinucleotides that is dinucleotide *ij*, and let *m*_*j*_*(u)* be the proportion of all the nucleotides that is nucleotide *j*. In our model the *m*_*j*_*(u)* and *d*_*ij*_*(u)* are related by:

(1) $$\mathrm{mj}\left(u\right)=\sum_{i}\mathrm{dij}\left(u\right)=\sum_{i}\mathrm{dji}\left(u\right)$$

The first sum is over all the dinucleotides *ij* where *j* is the downstream nucleotide, and the second sum is over all dinucleotides *ji* where *j* is the upstream nucleotide. The reason that these are the same is that in our model we assume an arbitrary long RNA or DNA sequence so every nucleotide occurs once in a dinucleotide as its upstream nucleotide and once in a dinucleotide as its downstream nucleotide. Each time there is a transition in our model the change affects equally the nucleotide where it is the upstream nucleotide and the dinucleotide where it is the downstream nucleotide. Hence provided the two sums are the same at the start of the simulation they will remain the same throughout.

Let *t*_*ijk*_*(u)* be the proportion at time *u* of all trinucleotides that is *ijk*. The trinucleotide *ijk* consists of an upstream dinucleotide *ij* and a downstream dinucleotide *jk* sharing a common middle nucleotide *j.* Following previous approaches [14] we assume that in trinucleotides the up and downstream dinucleotides that share a common middle nucleotide are independent. From this it follows that *t*_*ijk*_*(u)* = *d*_*ij*_*(u) P*_*jk|j*_*(u),* where *P*_*jk|j*_*(u)* is the proportion at time *u* of dinucleotide *jk* among all the dinucleotides whose left nucleotide is *j.* Since *P*_*jk|j*_*(u)= d*_*jk*_*(u)/ m*_*j*_*(u),* it follows that:

(2) $$\mathrm{tijk}\left(u\right)=\frac{\mathrm{dij}\left(u\right)\mathrm{djk}\left(u\right)}{\mathrm{mj}\left(u\right)}$$

The rate of change in the proportion of dinucleotide *ij* is given by the equation:

(3) $$\frac{\mathrm{ddxy}}{\mathrm{du}}\left(u\right)=\sum_{i,j,k}t_{\mathrm{ijk}}\left(u\right)\sum_{l}r\left(i,j\to l,k\right)b\left(\left(x,y\right),\left(i,j\to l,k\right)\right)$$

where *b((x, y), (i, j→l, k)))* is the change in the number of dinucleotides *xy* when a trinucleotide *ijk* turns to a trinucleotide *ilk*, *i.e*., the number of dinucleotides *xy* in trinucleotide *ilk* minus the number in trinucleotide *ijk*. For example, *b*((C, A), (C, A→T, G)) = −1 since one CpA is lost by changing from CAG to CTG. Also *b*((T, T), (T, C→T, T)) = 2, and *b*((A, A), (G, G→T, C)) = 0. It is not difficult to recognize that the value *b*((*x*, *y*), (*i*, *j* → *m*, *k*))) can take is − 2, − 1, 0, 1 or 2.

Let *F* denote the vector consisting of *κ* and all the factors *f.* For any given values of *θ* and *F*, the steady state dinucleotide proportions can be found by substituting (1) and (2) into (3) and integrating the resulting 16 nonlinear equations from an arbitrary starting composition until the proportions stabilize. Let *d*_*XY*_ be the limiting proportion of dinucleotide *XY* and let *m*_*X*_ be the limiting proportion of nucleotide *X*. The limiting C+G proportion, *ω* equals *m*_*C*_ + *m*_*G*_ and for each dinucleotide XY we can calculate the model’s prediction of the observed to expected dinucleotide ratio, XpYo/e, from *d*_*XY*_*/(m*_*X*_*m*_*Y*_*).* (If there was no correlation between the nucleotides in dinucleotides then this ratio would be 1.) By tabulating C+G and the resulting XpYo/e for a range of values *θ* and interpolating we can find for any value *ω* of C+G, the model’s estimate of XpYo/e. We denote this function by *M*_*XY*_*(ω,F)*.

To assess how good a fit our model is to a set of samples, the root mean square error, RMS, between the model and the data was calculated. Assume there are *N* samples and sample *n* has a C+G proportion of *ω*_*n*_ and the o/e ratio of the XY dinucleotide is *R*_*n,xy*_. Then for a vector of parameters *F* the RMS is:

$$\mathrm{RMS}\left(F\right)\;=\;\sqrt{\frac{\sum_{n=1}^{N}\sum_{x,y}{\left(R_{n,\mathrm{xy}}-M_{\mathrm{xy}}\left(\omega_{n},F\right)\right)}^{2}}{16N}}$$

The goal is to find the minimum value of RMS(*F*) and the corresponding value of the parameters *F,* which gives the best fit to the data. However we are interested in solutions in which only a small number of the factors, *f*, deviate from their default value of 1. The calculation is done in a series of stages. First we find the minimum values of RMS(*F*) for *κ* and each single factor in turn, and choose the factor that gives the best fit. Then we repeat the calculation allowing the values of *κ* and the previously selected factor and each other factor in turn to vary from their default values, and find which other factor allows the best reduction in RMS value. Then this is repeated to select at each step the best additional factor to add to the previously selected ones allowing at every step the re-optimisation of the values of *κ* and *f* for each of the previously selected nucleotides.

The steps are shown below. Here *M* is the total number of parameters to vary (*i.e.* either 49 or 97) and Max_K is the maximum number of parameters we want to allow to deviate from their default values. (In the DNA case the remaining parameters are set equal to their complementary parameter.) We number the parameters *f*_*0*_ = *κ, f*_*1*_ = *f*(A, A→C ), *f*_*2*_ = *f*(A, A→G ), … Variable R^Opt^ denotes the minimum value of RMS found when only the parameters in **D** are allowed to vary, and F^Opt^ the corresponding vector of parameter values.

Although the mutation rate *f(W→X, Y)* is a separate parameter in the model from *f(X→W, Y)*, their effects are related: setting *f(W→X, Y)* equal to a value *v* usually has a similar effect on the equilibrium compositions to setting *f(X→W, Y)* equal to the value 1*/v*. Consequently when presenting the results only the factor greater than 1 is reported**.**

### Strand symmetry in DNA sequences

Models used and the information that can be obtained from modelling mutational biases and selection pressure depends on the nature of the nucleic acid. Studies to date have been performed on genomic DNA; without evident polarity in its replication in eukaryotes, the actual number of independent dinucleotides amounts to only 10. These are ApT, TpA, CpG and GpC (self-complementary dinucleotides) and the following pairs which are present in equal frequencies in a large enough sequence sample; ApA and UpU, GpG/CpC, CpA/UpG, ApC/GpU, GpA/UpC and ApG/CpU. As described above in the model description, this symmetry leads to mutational biases dependent on a downstream base being indistinguishable from a complementary bias dependent on an upstream base. Thus, the well characterised methylation-induced mutation, represented here as C→T,G is formally equivalent a complementary process on the opposite DNA strand, *i.e.* C,G→A. In the current study, DNA mutations are by convention generally presented in the former format.

### Modeling dinucleotide biases in single stranded (RNA) sequences

For RNA sequences, different considerations apply. Mutational biases originating from context-dependent mutational biases will typically be symmetrical if originating from biases in the underlying DNA sequence from which it was transcribed, or in an RNA virus sequences where the same RNA polymerase transcribes sense and antisense genomic sequences. On the other hand, mutational biases from RNA polymerase II that transcribes mRNA sequences and dinucleotide composition abnormalities originating from selection in the cytoplasm lead to asymmetries that need to be separately modelled. As described above, modelling of mutational / selection biases in RNA therefore considers each dinucleotide separately (*e.g.* the frequency of UpC does not necessarily equal the frequency of GpA and as described in the previous section, mutations occurring in both upstream and downstream dinucleotide contexts have to be modelled separately, creating a total of 96 instead of 48 model parameters.

## Availability of supporting data

(Additional file 2: Figure S1, Additional file 3: Figure S2 and Additional file 5: Figure S3) and (Additional file 1: Table S1, Additional file 4: Table S2, Additional file 6: Table S3 and Additional file 9: Table S4) are available from:

## Abbreviations

RMS: root mean square; Κ: Transition / transversion ratios; (SNP): Single nucleotide polymorphism; θ: Proportion of G+C nucleotides.

## Competing interests

All four authors of the manuscript declare no competing interests.

## Authors’ contributions

PS and KMcK conceived the study. KMcK and WX developed the program to model dinucleotide frequencies. KB provided and analysed the datasets of genomic, and mRNA datasets and data for the SNP analysis. PS wrote the paper. All authors read and approved the final manuscript.

## Supplementary Material

Additional file 1: Table S1 Significance testing of differences in cpg and upa frequencies. Click here for file

Additional file 2: Figure S1 Observed / expected frequencies of all 16 dinucleotides in human DNA (Additional file 2: Figure S1A) and mRNA sequences (Additional file 2: Figure S1B), *D. rerio* DNA and mRNA sequences (S1C, S1D) and *A. gambiae* DNA and mRNA sequences (S1E, S1F). Values (y-axis) were plotted as a function of G+C content (x-axis). Frequencies of each dinucleotide predicted from mutational models with 1, 2 and 4 parameters (1p, 2p and 4p; see inset key) are superimposed on each distribution along with the quadratic line of best fit for each dataset generated from starting sequences ranging in G+C composition from 20%-80%. Click here for file

Additional file 3: Figure S2 Relationship between fragment length and modelled RMS scores of human DNA fragments of different lengths using 4 parameters. (A) Fragment lengths depicted in a linear scale. (B) To estimate RMS distances for sequences without sampling error (*ie*. for sequences of infinite length), sequence lengths were transformed using the empirically derived transformation 1/length^0.42^ to generate a linear relationship with RMS distances. The intercept with the y-axis line represents the RMS score for sequences of infinite length (0.0095). This represents the model error for this dataset. Click here for file

Additional file 4: Table S2 Measured and predicted minimum rms scores for dna and mrna datasets from different organisms. Click here for file

Additional file 5: Figure S3 Uncorrected model errors (y-axis) using mutational models with between 1 to 4 context-dependent mutational biases (labelled under graph line) formatted as in Figure 3. Click here for file

Additional file 6: Table S3 Reproducibility of corrected model error and mutational rates on data re-sampling. Click here for file

Additional file 7: Figure S4 Observed / expected frequencies of all 16 dinucleotides of mammalian viral RNA sequences (see legend to Additional file 2: Figure S1). Click here for file

Additional file 8: Figure S5 Uncorrected model errors (y-axis) for mammalian RNA viruses using mutational models with between 1 to 4 context-dependent mutational biases formatted as in Figure 3. Click here for file

Additional file 9: Table S4 Listing of mammalian viral sequences analysed in study. Click here for file

## References

[B1] Russell GJ Walker PM Elton RA Subak-Sharpe JH Doublet frequency analysis of fractionated vertebrate nuclear DNA J Mol Biol 1976 14 1 23 10.1016/S0022-2836(76)80090-3 1003479

[B2] Schwarz MN Trautner TA Kornberg A Enzymatic synthesis of deoxyribonucleic acid: XI: further studies on nearest neighbor base sequences in deoxyribonucleic acids J Biol Chem 1962 14 1961 1967 13918810

[B3] Josse J Kaiser AD Kornberg A Enzymatic synthesis of deoxyribonucleic acid: VIII: frequencies of nearest neighbor base sequences in deoxyribonucleic acid J Biol Chem 1961 14 864 875 13790780

[B4] Bird AP DNA methylation and the frequency of CpG in animal DNA Nucleic Acids Res 1980 14 1499 1504 10.1093/nar/8.7.1499 6253938 PMC324012

[B5] Coulondre C Miller JH Farabaugh PJ Gilbert W Molecular basis of base substitution hotspots in Escherichia coli Nature 1978 14 775 780 10.1038/274775a0 355893

[B6] Duan J Antezana MA Mammalian mutation pressure, synonymous codon choice, and mRNA degradation J Mol Evol 2003 14 694 701 10.1007/s00239-003-2519-1 14745538

[B7] Beutler E Gelbart T Han JH Koziol JA Beutler B Evolution of the genome and the genetic code: selection at the dinucleotide level by methylation and polyribonucleotide cleavage Proc Natl Acad Sci USA 1989 14 192 196 10.1073/pnas.86.1.192 2463621 PMC286430

[B8] Fryxell KJ Moon WJ CpG mutation rates in the human genome are highly dependent on local GC content Mol Biol Evol 2005 14 650 658 15537806 10.1093/molbev/msi043

[B9] Fryxell KJ Zuckerkandl E Cytosine deamination plays a primary role in the evolution of mammalian isochores Mol Biol Evol 2000 14 1371 1383 10.1093/oxfordjournals.molbev.a026420 10958853

[B10] Rima BK McFerran NV Dinucleotide and stop codon frequencies in single-stranded RNA viruses J Gen Virol 1997 14 2859 2870 9367373 10.1099/0022-1317-78-11-2859

[B11] Karlin S Doerfler W Cardon LR Why is CpG suppressed in the genomes of virtually all small eukaryotic viruses but not in those of large eukaryotic viruses? J Virol 1994 14 2889 2897 8151759 10.1128/jvi.68.5.2889-2897.1994 PMC236777

[B12] Belalov IS Lukashev AN Causes and implications of codon usage bias in RNA viruses PLoS One 2013 14 e56642 10.1371/journal.pone.0056642 23451064 PMC3581513

[B13] Simmen MW Genome-scale relationships between cytosine methylation and dinucleotide abundances in animals Genomics 2008 14 33 40 10.1016/j.ygeno.2008.03.009 18485662

[B14] Duret L Galtier N The covariation between TpA deficiency, CpG deficiency, and G+C content of human isochores is due to a mathematical artifact Mol Biol Evol 2000 14 1620 1625 10.1093/oxfordjournals.molbev.a026261 11070050

[B15] Lyko F Foret S Kucharski R Wolf S Falckenhayn C Maleszka R The honey bee epigenomes: differential methylation of brain DNA in queens and workers PLoS Biol 2010 14 e1000506 10.1371/journal.pbio.1000506 21072239 PMC2970541

[B16] Glastad KM Hunt BG Yi SV Goodisman MA DNA methylation in insects: on the brink of the epigenomic era Insect Mol Biol 2011 14 553 565 10.1111/j.1365-2583.2011.01092.x 21699596

[B17] Gruenbaum Y Naveh-Many T Cedar H Razin A Sequence specificity of methylation in higher plant DNA Nature 1981 14 860 862 10.1038/292860a0 6267477

[B18] Jabbari K Bernardi G Cytosine methylation and CpG, TpG (CpA) and TpA frequencies Gene 2004 14 143 149 15177689 10.1016/j.gene.2004.02.043

[B19] Lindsay H Yap VB Ying H Huttley GA Pitfalls of the most commonly used models of context dependent substitution Biol Direct 2008 14 52 10.1186/1745-6150-3-52 19087239 PMC2628887

[B20] Zhang W Bouffard GG Wallace SS Bond JP Estimation of DNA sequence context-dependent mutation rates using primate genomic sequences J Mol Evol 2007 14 207 214 10.1007/s00239-007-9000-5 17676366

[B21] Berard J Gueguen L Accurate estimation of substitution rates with neighbor-dependent models in a phylogenetic context Syst Biol 2012 14 510 521 10.1093/sysbio/sys024 22331438

[B22] Sved J Bird A The expected equilibrium of the CpG dinucleotide in vertebrate genomes under a mutation model Proc Natl Acad Sci USA 1990 14 4692 4696 10.1073/pnas.87.12.4692 2352943 PMC54183

[B23] Aissani B Bernardi G CpG islands, genes and isochores in the genomes of vertebrates Gene 1991 14 185 195 10.1016/0378-1119(91)90198-K 1937049

[B24] Shioiri C Takahata N Skew of mononucleotide frequencies, relative abundance of dinucleotides, and DNA strand asymmetry J Mol Evol 2001 14 364 376 10.1007/s002390010226 11675596

[B25] Tomso DJ Bell DA Sequence context at human single nucleotide polymorphisms: overrepresentation of CpG dinucleotide at polymorphic sites and suppression of variation in CpG islands J Mol Biol 2003 14 303 308 10.1016/S0022-2836(03)00120-7 12628237

[B26] Zhao Z Zhang F Sequence context analysis in the mouse genome: single nucleotide polymorphisms and CpG island sequences Genomics 2006 14 68 74 10.1016/j.ygeno.2005.09.012 16316740

[B27] Qiu L Moreira A Kaplan G Levitz R Wang JY Xu C Drlica K Degradation of hammerhead ribozymes by human ribonucleases Mol Gen Genet 1998 14 352 362 10.1007/s004380050741 9648739

[B28] Lobo FP Mota BE Pena SD Azevedo V Macedo AM Tauch A Machado CR Franco GR Virus-host coevolution: common patterns of nucleotide motif usage in Flaviviridae and their hosts PLoS ONE 2009 14 e6282 10.1371/journal.pone.0006282 19617912 PMC2707012

[B29] Koning FA Goujon C Bauby H Malim MH Target cell-mediated editing of HIV-1 cDNA by APOBEC3 proteins in human macrophages J Virol 2011 14 13448 13452 10.1128/JVI.00775-11 21957290 PMC3233168

[B30] Samuel CE Adenosine deaminases acting on RNA (ADARs) are both antiviral and proviral Virology 2011 14 180 193 10.1016/j.virol.2010.12.004 21211811 PMC3057271

[B31] Greenbaum BD Rabadan R Levine AJ Patterns of oligonucleotide sequences in viral and host cell RNA identify mediators of the host innate immune system PLoS ONE 2009 14 e5969 10.1371/journal.pone.0005969 19536338 PMC2694999

[B32] Greenbaum BD Levine AJ Bhanot G Rabadan R Patterns of evolution and host gene mimicry in influenza and other RNA viruses PLoS Pathog 2008 14 e1000079 10.1371/journal.ppat.1000079 18535658 PMC2390760

[B33] Sugiyama T Gursel M Takeshita F Coban C Conover J Kaisho T Akira S Klinman DM Ishii KJ CpG RNA: identification of novel single-stranded RNA that stimulates human CD14+CD11c+ monocytes J Immunol 2005 14 2273 2279 15699162 10.4049/jimmunol.174.4.2273

[B34] Burns CC Campagnoli R Shaw J Vincent A Jorba J Kew O Genetic inactivation of poliovirus infectivity by increasing the frequencies of CpG and UpA dinucleotides within and across synonymous capsid region codons J Virol 2009 14 9957 9969 10.1128/JVI.00508-09 19605476 PMC2747992

[B35] Burns CC Shaw J Campagnoli R Jorba J Vincent A Quay J Kew O Modulation of poliovirus replicative fitness in HeLa cells by deoptimization of synonymous codon usage in the capsid region J Virol 2006 14 3259 3272 10.1128/JVI.80.7.3259-3272.2006 16537593 PMC1440415

[B36] Coleman JR Papamichail D Skiena S Futcher B Wimmer E Mueller S Virus attenuation by genome-scale changes in codon pair bias Science 2008 14 1784 1787 10.1126/science.1155761 18583614 PMC2754401

[B37] Simmonds P SSE: a nucleotide and amino acid sequence analysis platform BMC Res Notes 2012 14 50 10.1186/1756-0500-5-50 22264264 PMC3292810

