# Supplementary material for: Modelling mutational and selection pressures on dinucleotides in eukaryotic phyla –selection against CpG and UpA in cytoplasmically expressed RNA and in RNA viruses
Source: BMC Genomics. 2013 Sep 10;14:610. doi: 10.1186/1471-2164-14-610 (PMC3829696; doi:10.1186/1471-2164-14-610)
Supplement: Additional file 1: Table S1 — Significance testing of differences in cpg and upa frequencies. [file 1471-2164-14-610-S1.doc]

TABLE S1

SIGNIFICANCE TESTING OF DIFFERENCES IN

CpG AND UpA FREQUENCIES

A) Human genomic DNA and mRNA sequences

|  |  |  | **CpG** | | **TpA/UpA^3^** | |
| --- | --- | --- | --- | --- | --- | --- |
| **G+C^1^** | **Seq.^2^** | **n** | **Mean** | ***p*** | **Mean** | ***p*** |
| 40%-45% | DNA | 16324 | 0.233 | <10^-10^ | 0.728 | <10^-10^ |
|  | RNA | 1052 | 0.320 | <10^-10^ | 0.606 | <10^-10^ |
| 45%-50% | DNA | 6875 | 0.270 | <10^-10^ | 0.675 | <10^-10^ |
|  | RNA | 1079 | 0.358 | <10^-10^ | 0.562 | <10^-10^ |
| 50%-55% | DNA | 2935 | 0.307 | <10^-10^ | 0.621 | <10^-10^ |
|  | RNA | 829 | 0.422 | <10^-10^ | 0.504 | <10^-10^ |
| 55%-60% | DNA | 651 | 0.349 | <10^-10^ | 0.567 | <10^-10^ |
|  | RNA | 900 | 0.472 | <10^-10^ | 0.447 | <10^-10^ |

^1^ Sequences were binned into quartiles of G+C content as non-expressed DNA and mRNA sequences showed differences in their distributions of G+C compositions.

^2^ Sequence type: DNA: non-expressed genomic DNA; RNA: Sequences of expressed mRNAs

^3^ Students t test; *p* < 10^-10^ for differences between DNA and mRNA sequences were also determined using the Kruskal-Wallis non-parametric test

B) Viral RNA sequences

|  |  | **CpG** | | **TpA/UpA** | |
| --- | --- | --- | --- | --- | --- |
| **Virus genome type** | **n** | **Mean** | ***p*^1^** | **Mean** | ***P*^2^** |
| dsRNA | 85 | 0.836 | <10^-10^ | 0.790 | 4 x 10^-4^ |
| Others | 427 | 0.455 | <10^-10^ | 0.727 | 8 x 10^-7^ |

^1^ *p* < 10^-10^ by Kruskall Wallace non-parametric test

^2^ *p =* 4 x 10^-4^ by Kruskall Wallace non-parametric test
