# Supplementary figures and images for: Modelling mutational and selection pressures on dinucleotides in eukaryotic phyla –selection against CpG and UpA in cytoplasmically expressed RNA and in RNA viruses
Source: BMC Genomics. 2013 Sep 10;14:610. doi: 10.1186/1471-2164-14-610 (PMC3829696; doi:10.1186/1471-2164-14-610)

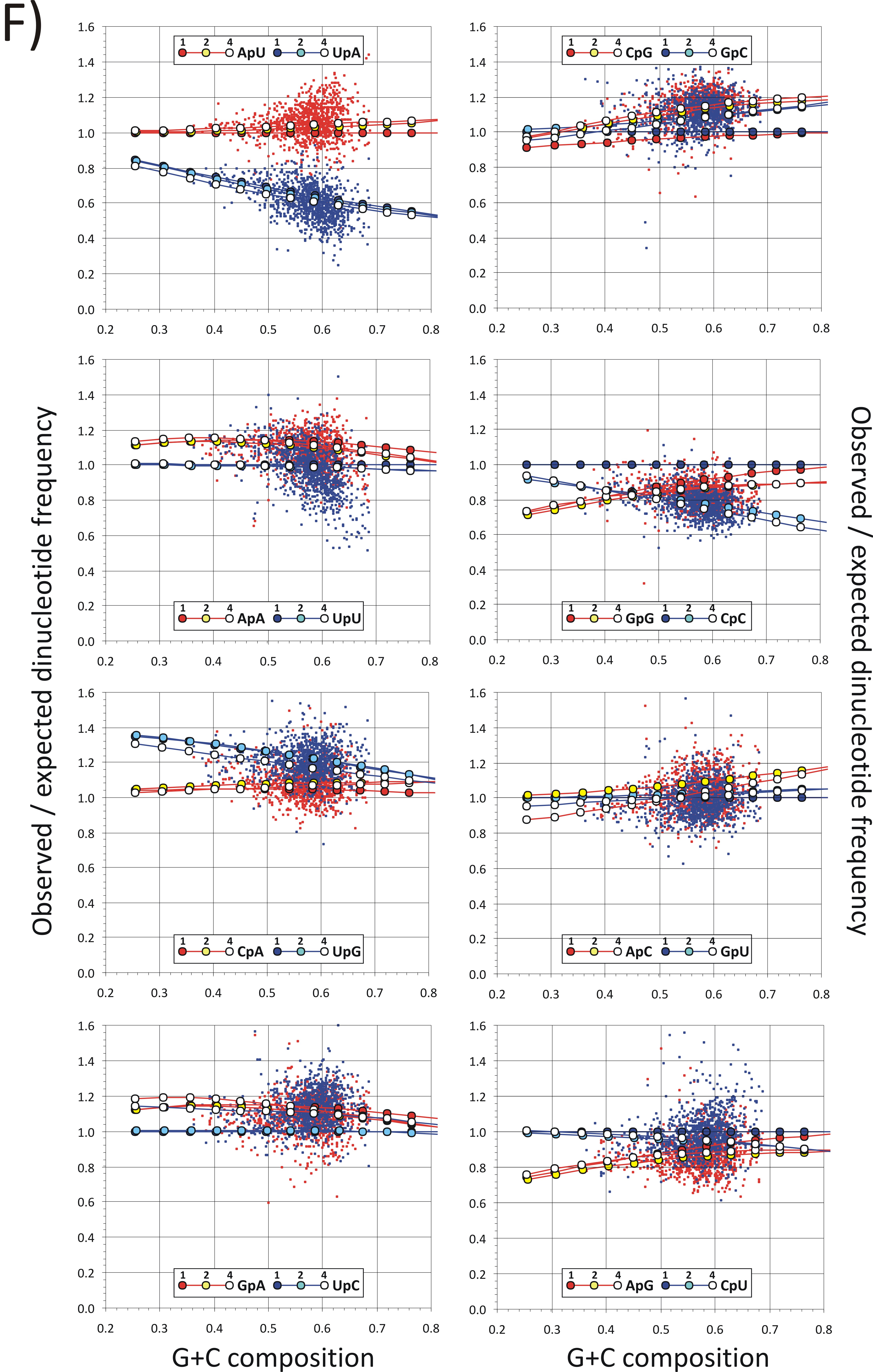

Supplement: Additional file 2: Figure S1 — Observed / expected frequencies of all 16 dinucleotides in human DNA (Additional file 2: Figure S1A) and mRNA sequences (Additional file 2: Figure S1B), D. rerio DNA and mRNA sequences (S1C, S1D) and A. gambiae DNA and mRNA sequences (S1E, S1F). Values (y-axis) were plotted as a function of G+C content (x-axis). Frequencies of each dinucleotide predicted from mutational models with 1, 2 and 4 parameters (1p, 2p and 4p; see inset key) are superimposed on each distribution along with the quadratic line of best fit for each dataset generated from starting sequences ranging in G+C composition from 20%-80%. [file 1471-2164-14-610-S2.zip › 6651377410224333_add10.jpeg]

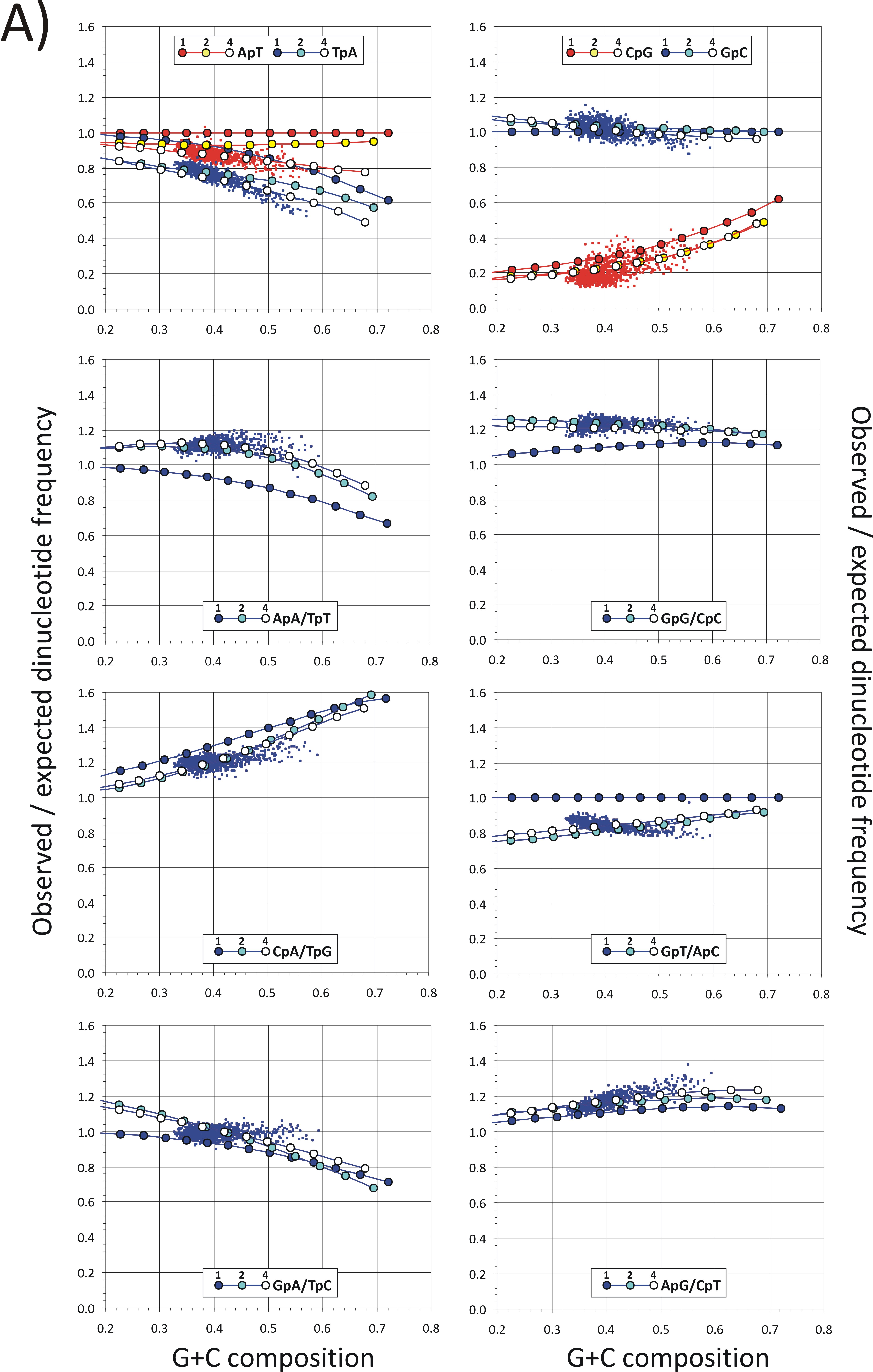

Supplement: Additional file 2: Figure S1 — Observed / expected frequencies of all 16 dinucleotides in human DNA (Additional file 2: Figure S1A) and mRNA sequences (Additional file 2: Figure S1B), D. rerio DNA and mRNA sequences (S1C, S1D) and A. gambiae DNA and mRNA sequences (S1E, S1F). Values (y-axis) were plotted as a function of G+C content (x-axis). Frequencies of each dinucleotide predicted from mutational models with 1, 2 and 4 parameters (1p, 2p and 4p; see inset key) are superimposed on each distribution along with the quadratic line of best fit for each dataset generated from starting sequences ranging in G+C composition from 20%-80%. [file 1471-2164-14-610-S2.zip › 6651377410224333_add5.jpeg]

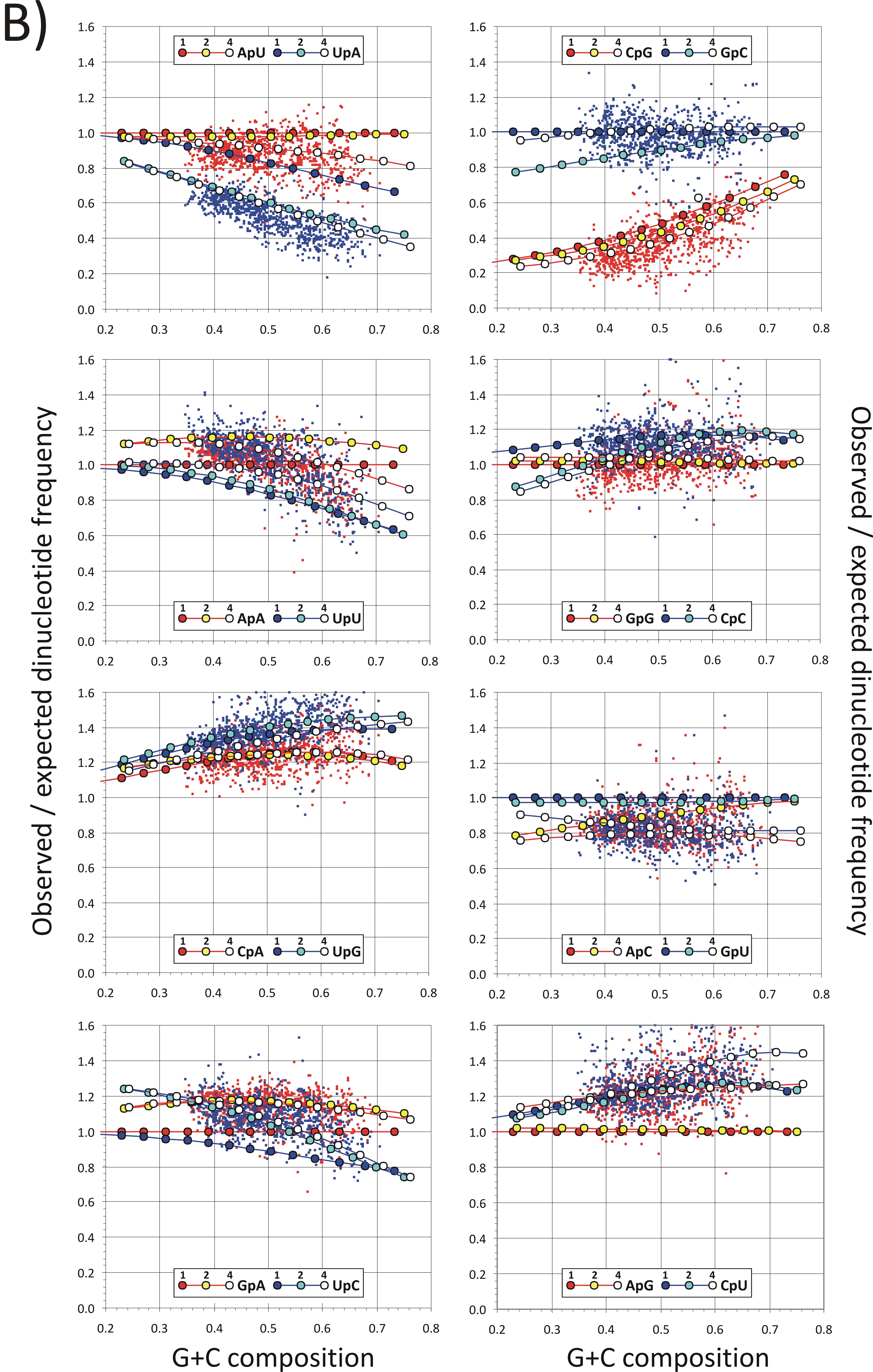

Supplement: Additional file 2: Figure S1 — Observed / expected frequencies of all 16 dinucleotides in human DNA (Additional file 2: Figure S1A) and mRNA sequences (Additional file 2: Figure S1B), D. rerio DNA and mRNA sequences (S1C, S1D) and A. gambiae DNA and mRNA sequences (S1E, S1F). Values (y-axis) were plotted as a function of G+C content (x-axis). Frequencies of each dinucleotide predicted from mutational models with 1, 2 and 4 parameters (1p, 2p and 4p; see inset key) are superimposed on each distribution along with the quadratic line of best fit for each dataset generated from starting sequences ranging in G+C composition from 20%-80%. [file 1471-2164-14-610-S2.zip › 6651377410224333_add6.jpeg]

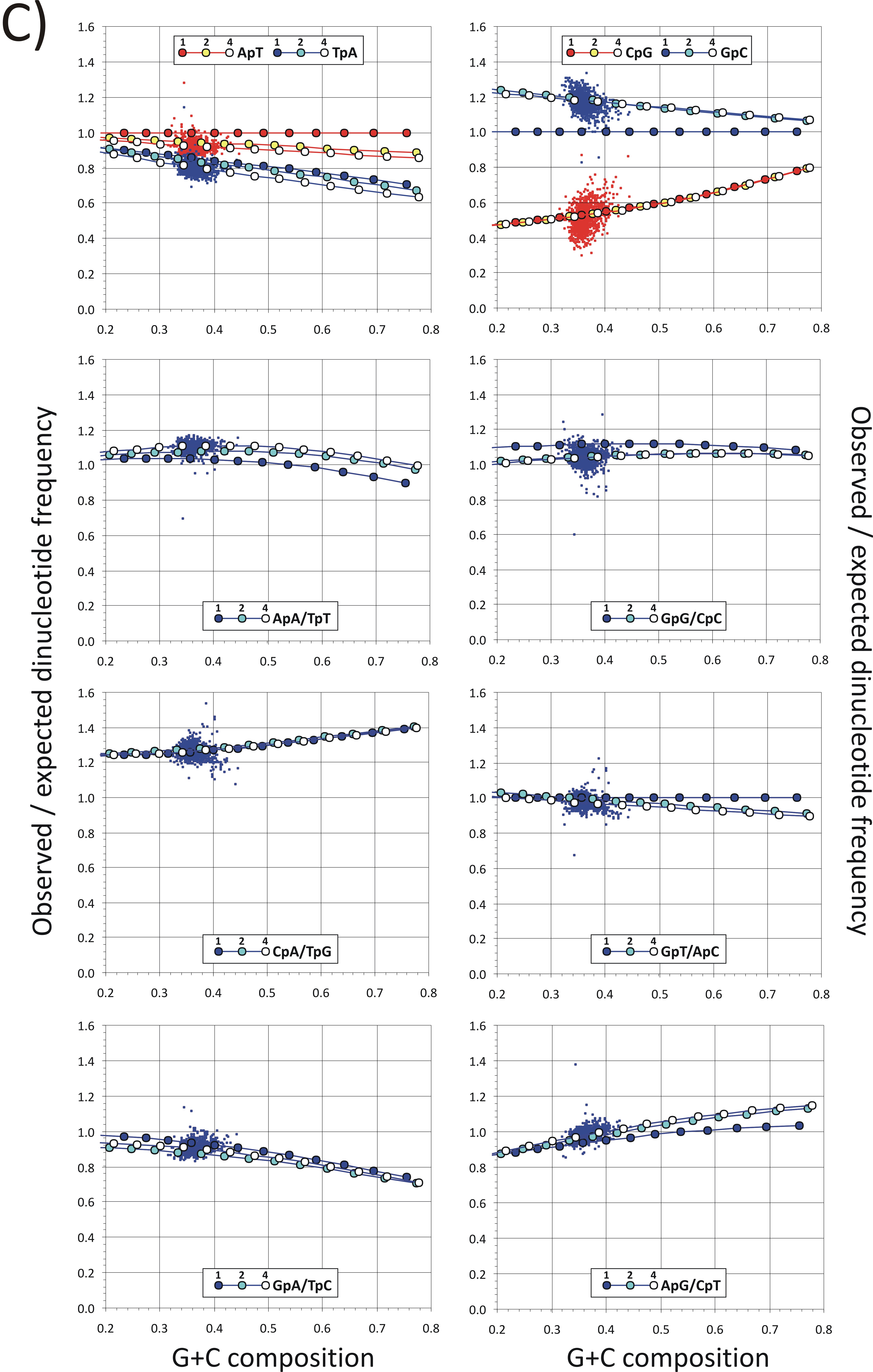

Supplement: Additional file 2: Figure S1 — Observed / expected frequencies of all 16 dinucleotides in human DNA (Additional file 2: Figure S1A) and mRNA sequences (Additional file 2: Figure S1B), D. rerio DNA and mRNA sequences (S1C, S1D) and A. gambiae DNA and mRNA sequences (S1E, S1F). Values (y-axis) were plotted as a function of G+C content (x-axis). Frequencies of each dinucleotide predicted from mutational models with 1, 2 and 4 parameters (1p, 2p and 4p; see inset key) are superimposed on each distribution along with the quadratic line of best fit for each dataset generated from starting sequences ranging in G+C composition from 20%-80%. [file 1471-2164-14-610-S2.zip › 6651377410224333_add7.jpeg]

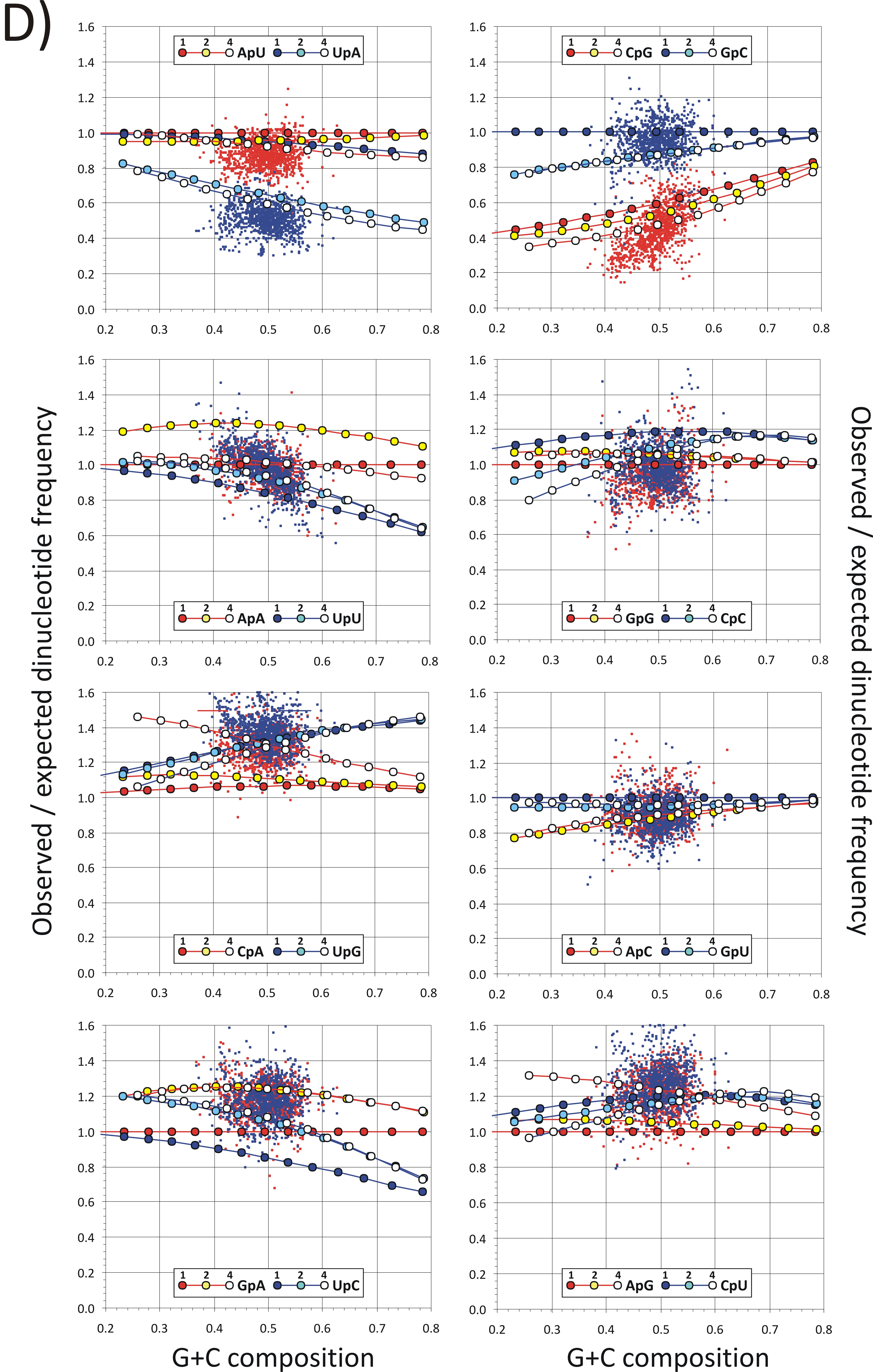

Supplement: Additional file 2: Figure S1 — Observed / expected frequencies of all 16 dinucleotides in human DNA (Additional file 2: Figure S1A) and mRNA sequences (Additional file 2: Figure S1B), D. rerio DNA and mRNA sequences (S1C, S1D) and A. gambiae DNA and mRNA sequences (S1E, S1F). Values (y-axis) were plotted as a function of G+C content (x-axis). Frequencies of each dinucleotide predicted from mutational models with 1, 2 and 4 parameters (1p, 2p and 4p; see inset key) are superimposed on each distribution along with the quadratic line of best fit for each dataset generated from starting sequences ranging in G+C composition from 20%-80%. [file 1471-2164-14-610-S2.zip › 6651377410224333_add8.jpeg]

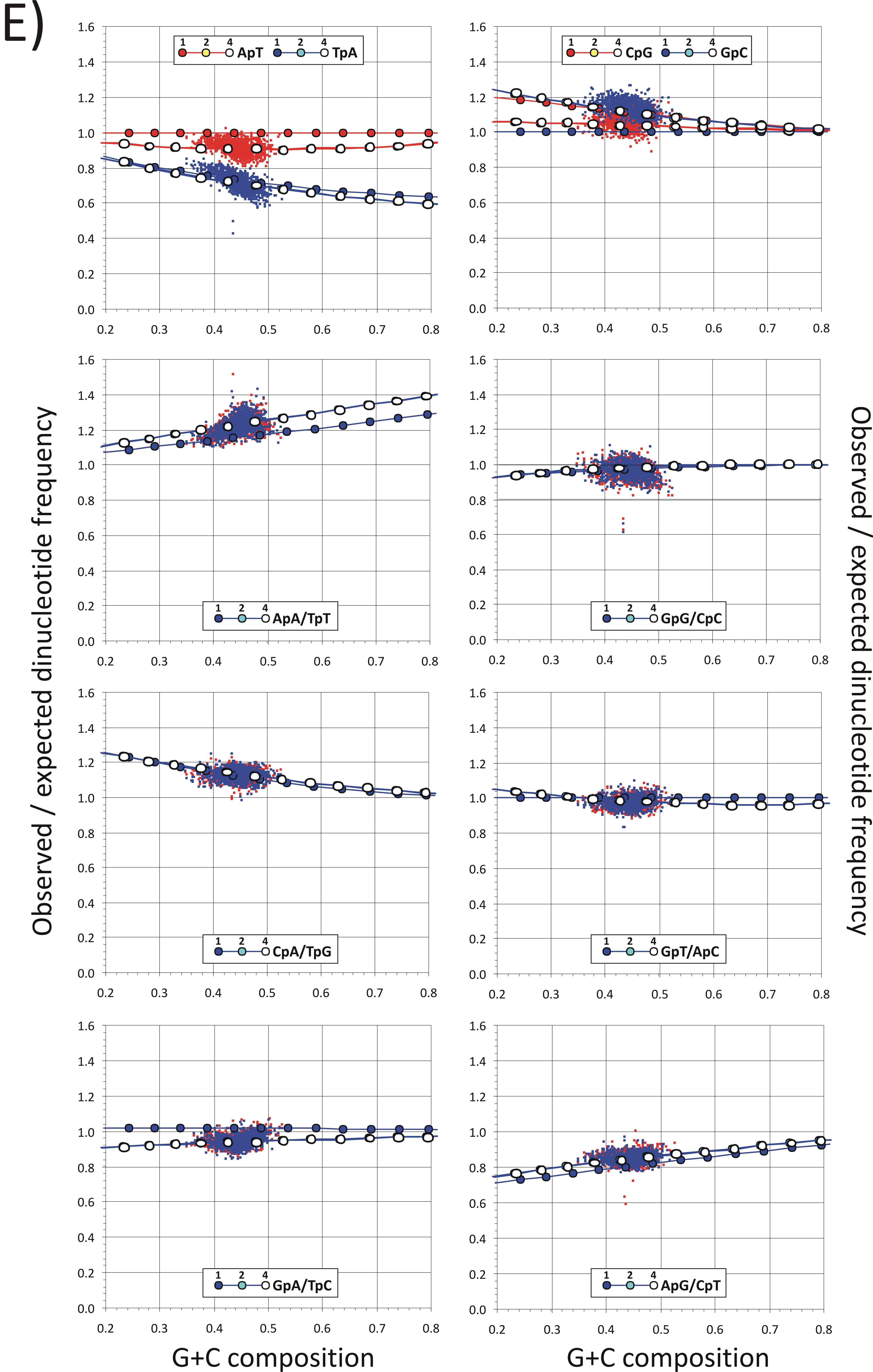

Supplement: Additional file 2: Figure S1 — Observed / expected frequencies of all 16 dinucleotides in human DNA (Additional file 2: Figure S1A) and mRNA sequences (Additional file 2: Figure S1B), D. rerio DNA and mRNA sequences (S1C, S1D) and A. gambiae DNA and mRNA sequences (S1E, S1F). Values (y-axis) were plotted as a function of G+C content (x-axis). Frequencies of each dinucleotide predicted from mutational models with 1, 2 and 4 parameters (1p, 2p and 4p; see inset key) are superimposed on each distribution along with the quadratic line of best fit for each dataset generated from starting sequences ranging in G+C composition from 20%-80%. [file 1471-2164-14-610-S2.zip › 6651377410224333_add9.jpeg]

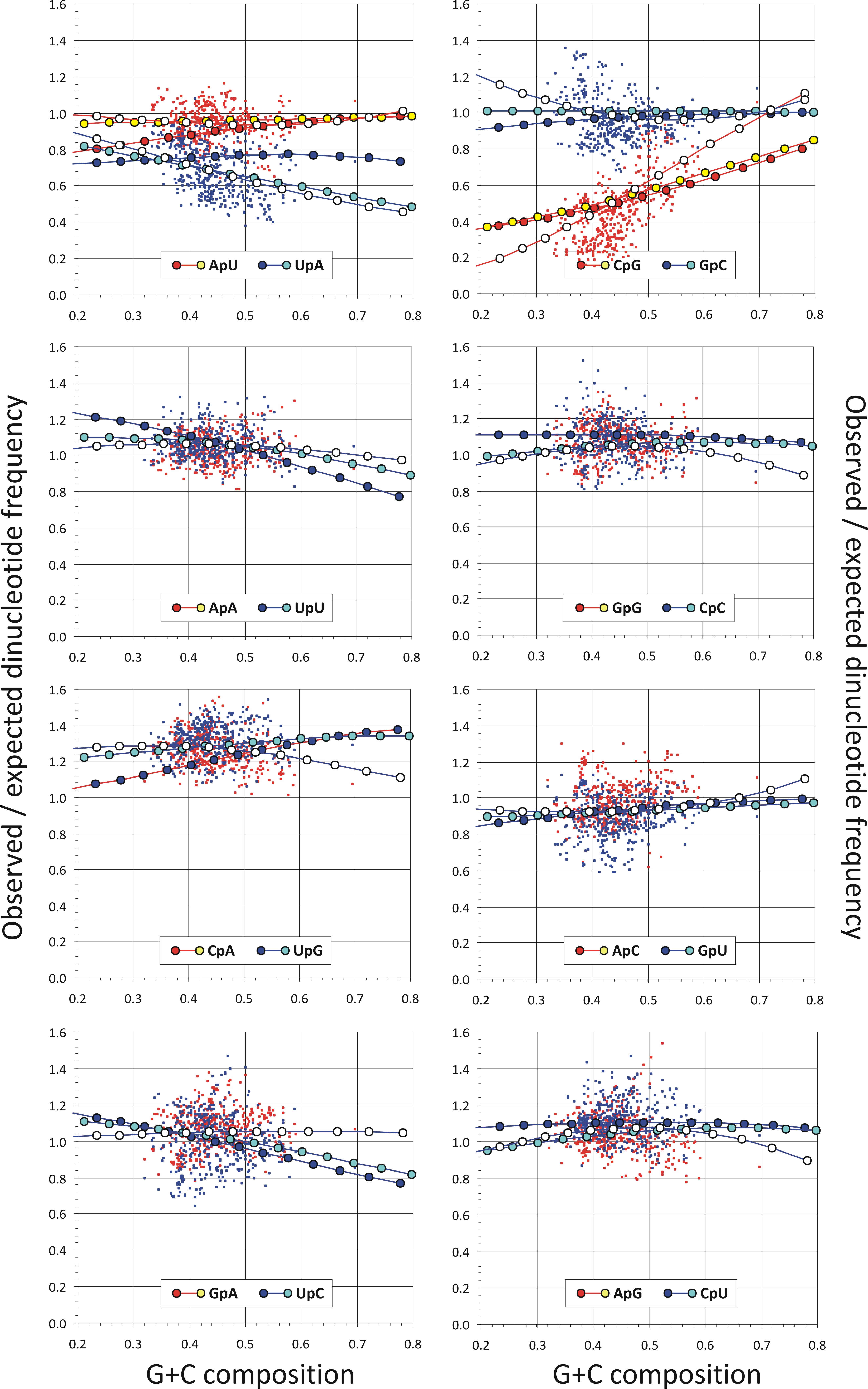

Supplement: Additional file 3: Figure S2 — Relationship between fragment length and modelled RMS scores of human DNA fragments of different lengths using 4 parameters. (A) Fragment lengths depicted in a linear scale. (B) To estimate RMS distances for sequences without sampling error (ie. for sequences of infinite length), sequence lengths were transformed using the empirically derived transformation 1/length0.42 to generate a linear relationship with RMS distances. The intercept with the y-axis line represents the RMS score for sequences of infinite length (0.0095). This represents the model error for this dataset. [file 1471-2164-14-610-S3.jpeg]

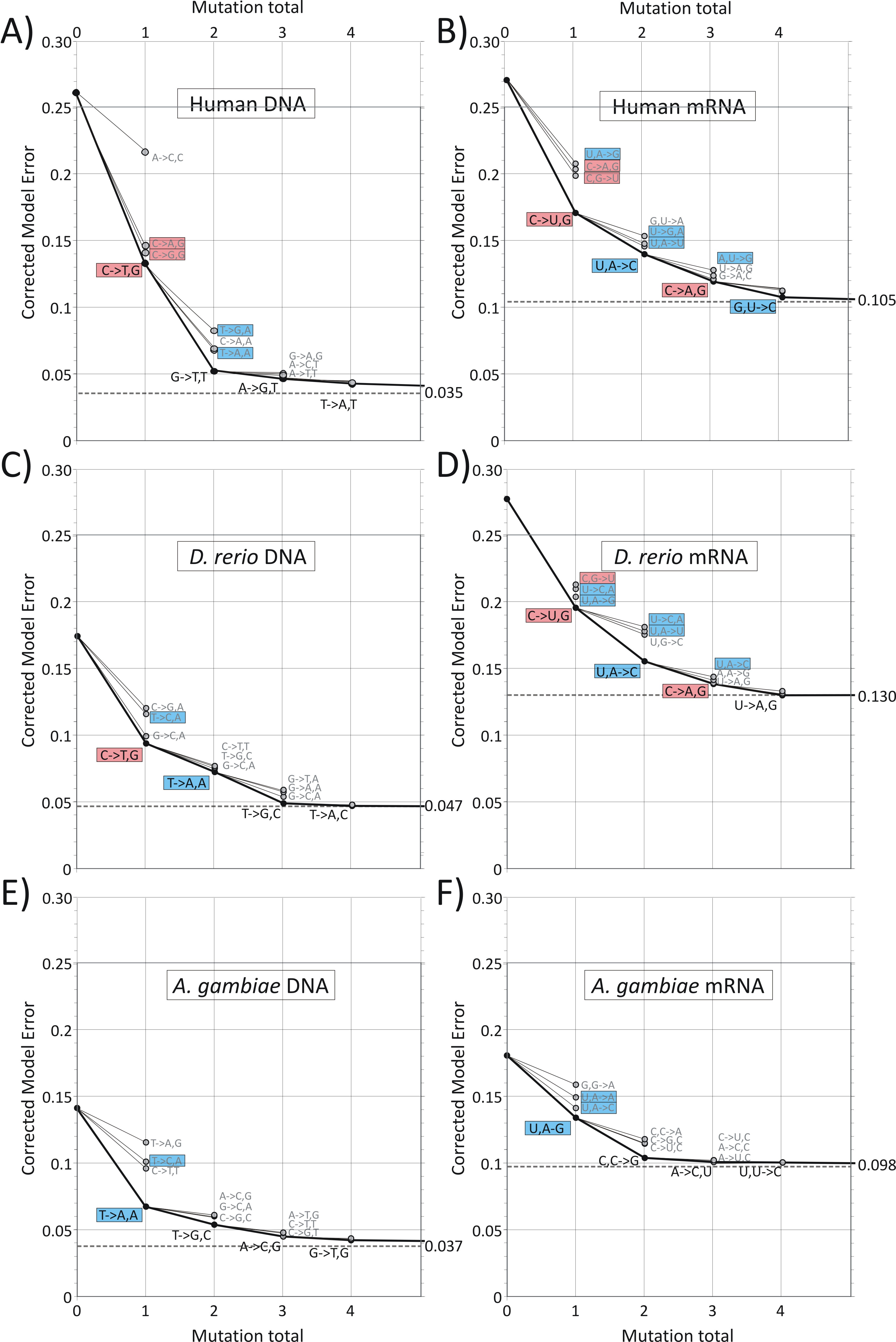

Supplement: Additional file 5: Figure S3 — Uncorrected model errors (y-axis) using mutational models with between 1 to 4 context-dependent mutational biases (labelled under graph line) formatted as in Figure 3. [file 1471-2164-14-610-S5.jpeg]

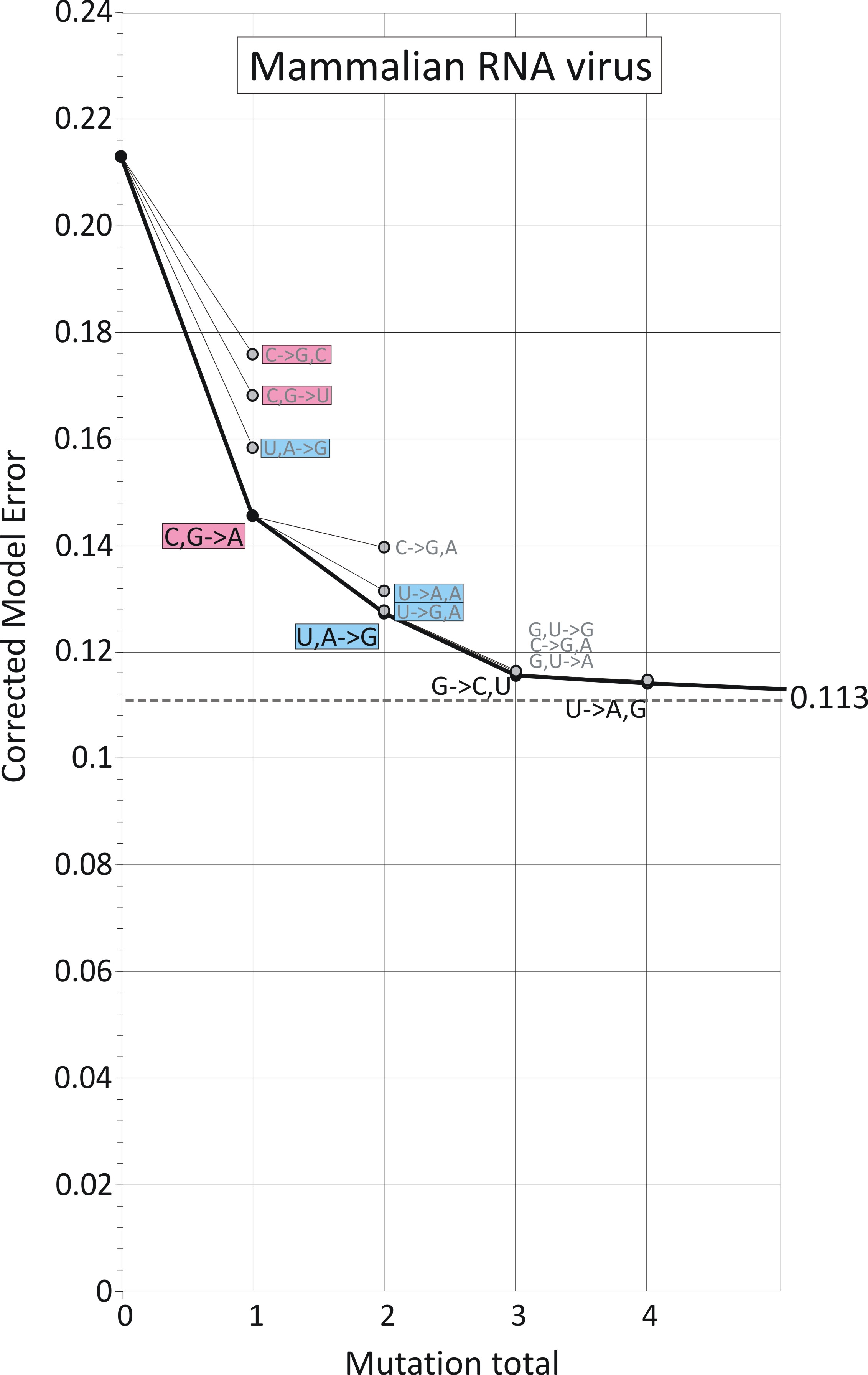

Supplement: Additional file 8: Figure S5 — Uncorrected model errors (y-axis) for mammalian RNA viruses using mutational models with between 1 to 4 context-dependent mutational biases formatted as in Figure 3. [file 1471-2164-14-610-S8.jpeg]
