# Supplementary material for: Modelling mutational and selection pressures on dinucleotides in eukaryotic phyla –selection against CpG and UpA in cytoplasmically expressed RNA and in RNA viruses
Source: BMC Genomics. 2013 Sep 10;14:610. doi: 10.1186/1471-2164-14-610 (PMC3829696; doi:10.1186/1471-2164-14-610)
Supplement: Additional file 4: Table S2 — Measured and predicted minimum rms scores for dna and mrna datasets from different organisms. [file 1471-2164-14-610-S4.doc]

TABLE S2

| **Species** | **Sequence** | **Measured^1^** | **Predicted^2^** |
| --- | --- | --- | --- |
| *H. sapiens* | DNA | 0.02747 | 0.032881 |
|  | mRNA | 0.097611 | 0.099367 |
| *M. musculus* | DNA | 0.029304 | 0.032881 |
|  | mRNA | 0.092483 | 0.096842 |
| *P. troglodytes* | DNA | 0.02651 | 0.032881 |
|  | mRNA | 0.096063 | 0.094497 |
| *A. gambiae* | DNA | 0.033899 | 0.032881 |
|  | mRNA | 0.096025 | 0.077089 |
| *D. rerio* | DNA | 0.040148 | 0.032881 |
|  | mRNA | 0.108158 | 0.084074 |
|  |  |  |  |
| Vert. RNA virus | Viral RNA | 0.111816 | 0.062658 |

MEASURED AND PREDICTED MINIMUM RMS SCORES

FOR DNA AND mRNA DATASETS FROM DIFFERENT ORGANISMS

^1^RMS scores derived from quadratic lines of best fit through observational data

^2^Contribution to RMS score from stochastic error using the empirically derived relationship (2.2 / length^0.42^) + 0.0095
