# Supplementary material for: Modelling mutational and selection pressures on dinucleotides in eukaryotic phyla –selection against CpG and UpA in cytoplasmically expressed RNA and in RNA viruses
Source: BMC Genomics. 2013 Sep 10;14:610. doi: 10.1186/1471-2164-14-610 (PMC3829696; doi:10.1186/1471-2164-14-610)
Supplement: Additional file 6: Table S3 — Reproducibility of corrected model error and mutational rates on data re-sampling. [file 1471-2164-14-610-S6.doc]

TABLE S3

REPRODUCIBILITY OF CORRECTED MODEL ERROR AND

MUTATIONAL RATES ON DATA RE-SAMPLING

| **Sample** | **Model Error** | **Ts/Tv** | **1^st^** | **Rate** | **2^nd^** | **Rate** | **3^rd^** | **Rate** |
| --- | --- | --- | --- | --- | --- | --- | --- | --- |
| 1 | 0.0135 | 3.1 | C→T,G | 12.06 | G→T,T | 6.16 | A→G,T | 1.42 |
| 2 | 0.0164 | 3.7 | C→T,G | 11.09 | G→T,T | 7.08 | A→G,T | 1.15 |
| 3 | 0.0179 | 3.4 | C→T,G | 11.50 | G→T,T | 7.15 | A→G,T | 1.30 |
| 4 | 0.0188 | 3.1 | C→T,G | 11.89 | G→T,T | 6.93 | A→G,T | 1.30 |
| SEM* | ±0.0012 | ±0.14 |  | ±0.216 |  | ±0.228 |  | ±0.055 |

*Standard error of the mean.
