# Supplementary material for: Modelling mutational and selection pressures on dinucleotides in eukaryotic phyla –selection against CpG and UpA in cytoplasmically expressed RNA and in RNA viruses
Source: BMC Genomics. 2013 Sep 10;14:610. doi: 10.1186/1471-2164-14-610 (PMC3829696; doi:10.1186/1471-2164-14-610)
Supplement: Additional file 9: Table S4 — Listing of mammalian viral sequences analysed in study. [file 1471-2164-14-610-S9.doc]

TABLE S4

LISTING OF MAMMALIAN VIRAL SEQUENCES ANALYSED IN STUDY

| **Nucleic acid** | **Refseq. No.** | **Accession no.** | **Virus descrition** | **Order** | **Family** |
| --- | --- | --- | --- | --- | --- |
| ssRNA- | NC_001498 | AB016162 | Measles virus | Mononegavirales | Paramyxoviridae |
| ssRNA- | NC_001542 | M13215 | Rabies virus | Mononegavirales | Rhabdoviridae |
| ssRNA- | NC_001552 | AB005795 | Sendai virus | Mononegavirales | Paramyxoviridae |
| ssRNA- | NC_001560 | J02428 | Vesicular stomatitis Indiana virus | Mononegavirales | Rhabdoviridae |
| ssRNA- | NC_001607 | U04608 | Borna disease virus | | Bornaviridae |
| ssRNA- | NC_001608 | DQ217792 | Lake Victoria marburgvirus - Musoke | Mononegavirales | Filoviridae |
| ssRNA- | NC_001781 | AF013254 | Human respiratory syncytial virus | Mononegavirales | Paramyxoviridae |
| ssRNA- | NC_001796 | AB012132 | Human parainfluenza virus 3 | Mononegavirales | Paramyxoviridae |
| ssRNA- | NC_001803 | U39661 | Respiratory syncytial virus | | Paramyxoviridae |
| ssRNA- | NC_001906 | AF017149 | Hendra virus | Mononegavirales | Paramyxoviridae |
| ssRNA- | NC_001921 | AF014953 | Canine distemper virus | Mononegavirales | Paramyxoviridae |
| ssRNA- | NC_001925 | X14383 | Bunyamwera virus L segment | | Bunyaviridae |
| ssRNA- | NC_001926 | M11852 | Bunyamwera virus M segment | | Bunyaviridae |
| ssRNA- | NC_001927 | D00353 | Bunyamwera virus segment S | | Bunyaviridae |
| ssRNA- | NC_001989 | AF092942 | Bovine respiratory syncytial virus | Mononegavirales | Paramyxoviridae |
| ssRNA- | NC_002016 | V01099 | Influenza A virus (A/Puerto Rico/8/34(H1N1)) segment 7 | | Orthomyxoviridae |
| ssRNA- | NC_002017 | V01088 | Influenza A virus (A/Puerto Rico/8/34(H1N1)) segment 4 | | Orthomyxoviridae |
| ssRNA- | NC_002018 | J02146 | Influenza A virus (A/Puerto Rico/8/34(H1N1)) segment 6 | | Orthomyxoviridae |
| ssRNA- | NC_002019 | J02147 | Influenza A virus (A/Puerto Rico/8/34(H1N1)) segment 5 | | Orthomyxoviridae |
| ssRNA- | NC_002020 | J02150 | Influenza A virus (A/Puerto Rico/8/34(H1N1)) segment 8 | | Orthomyxoviridae |
| ssRNA- | NC_002021 | J02151 | Influenza A virus (A/Puerto Rico/8/34(H1N1)) segment 2 | | Orthomyxoviridae |
| ssRNA- | NC_002022 | V01106 | Influenza A virus (A/Puerto Rico/8/34(H1N1)) segment 3 | | Orthomyxoviridae |
| ssRNA- | NC_002023 | V00603 | Influenza A virus (A/Puerto Rico/8/34(H1N1)) segment 1 | | Orthomyxoviridae |
| ssRNA- | NC_002043 | X56464 | Rift Valley fever virus L segment | | Bunyaviridae |
| ssRNA- | NC_002044 | M25276 | Rift Valley fever virus M segment | | Bunyaviridae |
| ssRNA- | NC_002045 | X53771 | Rift Valley fever virus segment S | | Bunyaviridae |
| ssRNA- | NC_002161 | AF178654 | Bovine parainfluenza virus 3 | Mononegavirales | Paramyxoviridae |
| ssRNA- | NC_002199 | AF079780 | Tupaia paramyxovirus | Mononegavirales | Paramyxoviridae |
| ssRNA- | NC_002200 | AB040874 | Mumps virus | Mononegavirales | Paramyxoviridae |
| ssRNA- | NC_002204 | M14880 | Influenza B virus RNA 1 | | Orthomyxoviridae |
| ssRNA- | NC_002205 | AF101982 | Influenza B virus RNA-2 | | Orthomyxoviridae |
| ssRNA- | NC_002206 | AF102017 | Influenza B virus RNA-3 | | Orthomyxoviridae |
| ssRNA- | NC_002207 | K00423 | Influenza B virus RNA 4 | | Orthomyxoviridae |
| ssRNA- | NC_002208 | K01395 | Influenza B virus RNA 5 | | Orthomyxoviridae |
| ssRNA- | NC_002209 | J02095 | Influenza B virus RNA 6 | | Orthomyxoviridae |
| ssRNA- | NC_002210 | J02094 | Influenza B virus RNA 7 | | Orthomyxoviridae |
| ssRNA- | NC_002211 | J02096 | Influenza B virus RNA 8 | | Orthomyxoviridae |
| ssRNA- | NC_002526 | AF234533 | Bovine ephemeral fever virus | Mononegavirales | Rhabdoviridae |
| ssRNA- | NC_002549 | AF086833 | Zaire ebolavirus | Mononegavirales | Filoviridae |
| ssRNA- | NC_002728 | AF212302 | Nipah virus | Mononegavirales | Paramyxoviridae |
| ssRNA- | NC_003243 | AF081020 | Australian bat lyssavirus | Mononegavirales | Rhabdoviridae |
| ssRNA- | NC_003443 | X57559 | Human parainfluenza virus 2 | Mononegavirales | Paramyxoviridae |
| ssRNA- | NC_003461 | AF457102 | Human parainfluenza virus 1 | Mononegavirales | Paramyxoviridae |
| ssRNA- | NC_003466 | AF291702 | Andes virus segment S | | Bunyaviridae |
| ssRNA- | NC_003467 | AF291703 | Andes virus segment M | | Bunyaviridae |
| ssRNA- | NC_003468 | AF291704 | Andes virus segment L | | Bunyaviridae |
| ssRNA- | NC_004074 | AF298895 | Tioman virus | Mononegavirales | Paramyxoviridae |
| ssRNA- | NC_004108 | AF528165 | La Crosse virus segment L | | Bunyaviridae |
| ssRNA- | NC_004109 | AF528166 | La Crosse virus segment M | | Bunyaviridae |
| ssRNA- | NC_004110 | AF528167 | La Crosse virus segment S | | Bunyaviridae |
| ssRNA- | NC_004148 | AY297749 | Human metapneumovirus | Mononegavirales | Paramyxoviridae |
| ssRNA- | NC_004157 | AF434161 | Dugbe virus segment S | | Bunyaviridae |
| ssRNA- | NC_004158 | M94133 | Dugbe virus segment M | | Bunyaviridae |
| ssRNA- | NC_004159 | U15018 | Dugbe virus segment L | | Bunyaviridae |
| ssRNA- | NC_004161 | AF522874 | Reston Ebola virus | Mononegavirales | Filoviridae |
| ssRNA- | NC_004291 | J04331 | Lymphocytic choriomeningitis virus segment L | | Arenaviridae |
| ssRNA- | NC_004292 | J04340 | Tacaribe virus segment L | | Arenaviridae |
| ssRNA- | NC_004293 | M20304 | Tacaribe virus segment S | | Arenaviridae |
| ssRNA- | NC_004294 | M20869 | Lymphocytic choriomeningitis virus segment S | | Arenaviridae |
| ssRNA- | NC_004296 | J04324 | Lassa virus segment S | | Arenaviridae |
| ssRNA- | NC_004297 | U73034 | Lassa virus segment L | | Arenaviridae |
| ssRNA- | NC_004905 | AJ289871 | Influenza A virus (A/Hong Kong/1073/99(H9N2)) | | Orthomyxoviridae |
| ssRNA- | NC_004906 | AJ278649 | Influenza A virus (A/Hong Kong/1073/99(H9N2)) segment 8 | | Orthomyxoviridae |
| ssRNA- | NC_004907 | AJ278646 | Influenza A virus (A/Hong Kong/1073/99(H9N2)) segment 7 | | Orthomyxoviridae |
| ssRNA- | NC_004908 | AJ404626 | Influenza A virus (A/Hong Kong/1073/99(H9N2)) segment 4 | | Orthomyxoviridae |
| ssRNA- | NC_004909 | AJ404629 | Influenza A virus (A/Hong Kong/1073/99(H9N2)) segment 6 | | Orthomyxoviridae |
| ssRNA- | NC_004910 | AJ404630 | Influenza A virus (A/Hong Kong/1073/99(H9N2)) segment 1 | | Orthomyxoviridae |
| ssRNA- | NC_004911 | AJ404634 | Influenza A virus (A/Hong Kong/1073/99(H9N2)) segment 2 | | Orthomyxoviridae |
| ssRNA- | NC_004912 | AJ404637 | Influenza A virus (A/Hong Kong/1073/99(H9N2)) segment 3 | | Orthomyxoviridae |
| ssRNA- | NC_005077 | AY129247 | Guanarito virus segment S | | Arenaviridae |
| ssRNA- | NC_005078 | AY129248 | Machupo virus segment S | | Arenaviridae |
| ssRNA- | NC_005079 | AY358021 | Machupo virus segment L | | Arenaviridae |
| ssRNA- | NC_005080 | AY358022 | Junin virus segment L | | Arenaviridae |
| ssRNA- | NC_005081 | AY358023 | Junin virus segment S | | Arenaviridae |
| ssRNA- | NC_005082 | AY358024 | Guanarito virus segment L | | Arenaviridae |
| ssRNA- | NC_005084 | AY141760 | Fer-de-lance virus | Mononegavirales | Paramyxoviridae |
| ssRNA- | NC_005214 | D10759 | Uukuniemi virus segment L | | Bunyaviridae |
| ssRNA- | NC_005215 | L25783 | Sin Nombre virus segment M | | Bunyaviridae |
| ssRNA- | NC_005216 | L25784 | Sin Nombre virus segment S | | Bunyaviridae |
| ssRNA- | NC_005217 | L37901 | Sin Nombre virus segment L | | Bunyaviridae |
| ssRNA- | NC_005218 | M14626 | Hantaan virus segment S | | Bunyaviridae |
| ssRNA- | NC_005219 | M14627 | Hantaan virus segment M | | Bunyaviridae |
| ssRNA- | NC_005220 | M17417 | Uukuniemi virus segment M | | Bunyaviridae |
| ssRNA- | NC_005221 | M33551 | Uukuniemi virus segment S | | Bunyaviridae |
| ssRNA- | NC_005222 | X55901 | Hantaan virus segment L | | Bunyaviridae |
| ssRNA- | NC_005223 | X61034 | Puumala virus segment M | | Bunyaviridae |
| ssRNA- | NC_005224 | X61035 | Puumala virus segment S | | Bunyaviridae |
| ssRNA- | NC_005225 | Z66548 | Puumala virus segment L | | Bunyaviridae |
| ssRNA- | NC_005226 | AJ005637 | Tula virus segment L | | Bunyaviridae |
| ssRNA- | NC_005227 | Z69991 | Tula virus segment S | | Bunyaviridae |
| ssRNA- | NC_005228 | Z69993 | Tula virus segment M | | Bunyaviridae |
| ssRNA- | NC_005233 | AJ410615 | Dobrava virus segment S | | Bunyaviridae |
| ssRNA- | NC_005234 | AJ410616 | Dobrava virus segment M | | Bunyaviridae |
| ssRNA- | NC_005235 | AJ410617 | Dobrava-Belgrade virus strain DOBV/Ano-Poroia/Afl9/1999 | | Bunyaviridae |
| ssRNA- | NC_005236 | AY273791 | Seoul virus strain 80-39 segment S | | Bunyaviridae |
| ssRNA- | NC_005237 | S47716 | Seoul virus segment M | | Bunyaviridae |
| ssRNA- | NC_005238 | X56492 | Seoul virus strain Seoul 80-39 clone 1 | | Bunyaviridae |
| ssRNA- | NC_005283 | AJ608288 | Dolphin morbillivirus | | Paramyxoviridae |
| ssRNA- | NC_005300 | AF467768 | Crimean-Congo hemorrhagic fever virus segment M | | Bunyaviridae |
| ssRNA- | NC_005301 | AY389361 | Crimean-Congo hemorrhagic fever virus segment L | | Bunyaviridae |
| ssRNA- | NC_005302 | U88410 | Crimean-Congo hemorrhagic fever virus segment S | | Bunyaviridae |
| ssRNA- | NC_005339 | AY286409 | Mossman virus | Mononegavirales | Paramyxoviridae |
| ssRNA- | NC_005775 | AF441119 | Oropouche virus segment M | | Bunyaviridae |
| ssRNA- | NC_005776 | AF484424 | Oropouche virus segment L | | Bunyaviridae |
| ssRNA- | NC_005777 | AY237111 | Oropouche virus segment S | | Bunyaviridae |
| ssRNA- | NC_005894 | AF485262 | Pirital virus segment S | | Arenaviridae |
| ssRNA- | NC_005897 | AY494081 | Pirital virus segment L | | Arenaviridae |
| ssRNA- | NC_006296 | X98291 | Rinderpest virus (strain Kabete O) | Mononegavirales | Paramyxoviridae |
| ssRNA- | NC_006306 | AB283001 | Influenza C virus (C/Ann Arbor/1/50) segment 7 | | Orthomyxoviridae |
| ssRNA- | NC_006307 | AB126191 | Influenza C virus segment 1, partial sequence | | Orthomyxoviridae |
| ssRNA- | NC_006308 | AB126192 | Influenza C virus (C/Ann Arbor/1/50) segment 2 | | Orthomyxoviridae |
| ssRNA- | NC_006309 | AB126193 | Influenza C virus (C/Ann Arbor/1/50) segment 3 | | Orthomyxoviridae |
| ssRNA- | NC_006310 | AB126194 | Influenza C virus (C/Ann Arbor/1/50) segment 4 | | Orthomyxoviridae |
| ssRNA- | NC_006311 | AB126195 | Influenza C virus (C/Ann Arbor/1/50) segment 5 | | Orthomyxoviridae |
| ssRNA- | NC_006312 | AB126196 | Influenza C virus (C/Ann Arbor/1/50) segment 6 | | Orthomyxoviridae |
| ssRNA- | NC_006313 | AY358026 | Sabia virus segment L | | Arenaviridae |
| ssRNA- | NC_006317 | U41071 | Sabia virus segment S | | Arenaviridae |
| ssRNA- | NC_006318 | X53794 | Toscana virus segment S | | Bunyaviridae |
| ssRNA- | NC_006319 | X68414 | Toscana virus segment L | | Bunyaviridae |
| ssRNA- | NC_006320 | X89628 | Toscana virus segment M | | Bunyaviridae |
| ssRNA- | NC_006383 | AJ849636 | Peste-des-petits-ruminants virus | Mononegavirales | Paramyxoviridae |
| ssRNA- | NC_006428 | X64275 | Simian virus 41 | Mononegavirales | Paramyxoviridae |
| ssRNA- | NC_006429 | Y09762 | Mokola virus | Mononegavirales | Rhabdoviridae |
| ssRNA- | NC_006430 | AF052755 | Simian virus 5 | Mononegavirales | Paramyxoviridae |
| ssRNA- | NC_006432 | AY729654 | Sudan ebolavirus | Mononegavirales | Filoviridae |
| ssRNA- | NC_006433 | AF184987 | Hantavirus Z10 segment S | | Bunyaviridae |
| ssRNA- | NC_006435 | AF189155 | Hantavirus Z10 segment L | | Bunyaviridae |
| ssRNA- | NC_006437 | AF276987 | Hantavirus Z10 segment M | | Bunyaviridae |
| ssRNA- | NC_006439 | AF427517 | Pichinde virus segment L | | Arenaviridae |
| ssRNA- | NC_006447 | K02734 | Pichinde virus segment S | | Arenaviridae |
| ssRNA- | NC_006495 | AF004985 | Thogoto virus segment 2 | | Orthomyxoviridae |
| ssRNA- | NC_006496 | AF006073 | Thogoto virus segment 3 | | Orthomyxoviridae |
| ssRNA- | NC_006504 | AF527531 | Thogoto virus segment 6 | | Orthomyxoviridae |
| ssRNA- | NC_006506 | M77280 | Thogoto virus segment 4 | | Orthomyxoviridae |
| ssRNA- | NC_006507 | X96872 | Thogoto virus segment 5 | | Orthomyxoviridae |
| ssRNA- | NC_006508 | Y17873 | Thogoto virus segment 1 | | Orthomyxoviridae |
| ssRNA- | NC_006572 | AY772167 | Mopeia Lassa reassortant 29 segment L | | Arenaviridae |
| ssRNA- | NC_006573 | AY772168 | Mopeia Lassa reassortant 29 segment S | | Arenaviridae |
| ssRNA- | NC_006574 | AY772169 | Mopeia virus AN20410 segment L | | Arenaviridae |
| ssRNA- | NC_006575 | AY772170 | Mopeia virus AN20410 segment S | | Arenaviridae |
| ssRNA- | NC_006579 | AY743909 | Pneumonia virus of mice J3666 | Mononegavirales | Paramyxoviridae |
| ssRNA- | NC_006942 | AY674964 | Taro vein chlorosis virus | Mononegavirales | Rhabdoviridae |
| ssRNA- | NC_007020 | AY840978 | Tupaia rhabdovirus | Mononegavirales | Rhabdoviridae |
| ssRNA- | NC_007366 | CY002064 | Influenza A virus (A/New York/392/2004(H3N2)) segment 4 | | Orthomyxoviridae |
| ssRNA- | NC_007367 | CY002065 | Influenza A virus (A/New York/392/2004(H3N2)) segment 7 | | Orthomyxoviridae |
| ssRNA- | NC_007368 | CY002066 | Influenza A virus (A/New York/392/2004(H3N2)) segment 6 | | Orthomyxoviridae |
| ssRNA- | NC_007369 | CY002067 | Influenza A virus (A/New York/392/2004(H3N2)) segment 5 | | Orthomyxoviridae |
| ssRNA- | NC_007370 | CY002068 | Influenza A virus (A/New York/392/2004(H3N2)) segment 8 | | Orthomyxoviridae |
| ssRNA- | NC_007371 | CY002069 | Influenza A virus (A/New York/392/2004(H3N2)) segment 3 | | Orthomyxoviridae |
| ssRNA- | NC_007372 | CY002070 | Influenza A virus (A/New York/392/2004(H3N2)) segment 2 | | Orthomyxoviridae |
| ssRNA- | NC_007373 | CY002071 | Influenza A virus (A/New York/392/2004(H3N2)) segment 1 | | Orthomyxoviridae |
| ssRNA- | NC_007374 | L11133 | Influenza A virus (A/Korea/426/68(H2N2)) segment 4 | | Orthomyxoviridae |
| ssRNA- | NC_007375 | M25935 | Influenza A virus (A/Korea/426/68(H2N2)) segment 2 | | Orthomyxoviridae |
| ssRNA- | NC_007376 | M26079 | Influenza A virus (A/Korea/426/68(H2N2)) segment 3 | | Orthomyxoviridae |
| ssRNA- | NC_007377 | M63531 | Influenza A virus (A/Korea/426/68(H2N2)) segment 7 | | Orthomyxoviridae |
| ssRNA- | NC_007378 | M73524 | Influenza A virus (A/Korea/426/68(H2N2)) segment 1 | | Orthomyxoviridae |
| ssRNA- | NC_007380 | AY210191 | Influenza A virus (A/Korea/426/68(H2N2)) segment 8 | | Orthomyxoviridae |
| ssRNA- | NC_007381 | AY210103 | Influenza A virus (A/Korea/426/68(H2N2)) segment 5 | | Orthomyxoviridae |
| ssRNA- | NC_007382 | AY209932 | Influenza A virus (A/Korea/426/68(H2N2)) segment 6 | | Orthomyxoviridae |
| ssRNA- | NC_007454 | AY900001 | J-virus | Mononegavirales | Paramyxoviridae |
| ssRNA- | NC_007620 | AF326114 | Menangle virus | Mononegavirales | Paramyxoviridae |
| ssRNA- | NC_007803 | DQ100461 | Beilong virus | Mononegavirales | Paramyxoviridae |
| ssRNA- | NC_007903 | AY342390 | Mobala virus segment S | | Arenaviridae |
| ssRNA- | NC_007904 | DQ328876 | Mobala virus segment L | | Arenaviridae |
| ssRNA- | NC_007905 | DQ328877 | Ippy virus segment S | | Arenaviridae |
| ssRNA- | NC_007906 | DQ328878 | Ippy virus segment L | | Arenaviridae |
| ssRNA- | NC_009489 | EF095490 | Mapuera virus | Mononegavirales | Paramyxoviridae |
| ssRNA- | NC_009527 | EF157976 | European bat lyssavirus 1 | Mononegavirales | Rhabdoviridae |
| ssRNA- | NC_009528 | EF157977 | European bat lyssavirus 2 | Mononegavirales | Rhabdoviridae |
| ssRNA- | NC_009640 | BK005918 | Porcine rubulavirus | Mononegavirales | Paramyxoviridae |
| ssRNA- | NC_009894 | AB190458 | Akabane virus segment L | | Bunyaviridae |
| ssRNA- | NC_009895 | AB100604 | Akabane virus segment M | | Bunyaviridae |
| ssRNA- | NC_009896 | AB000851 | Akabane virus segment S | | Bunyaviridae |
| ssRNA- | NC_010247 | AF485256 | Amapari virus segment S | | Arenaviridae |
| ssRNA- | NC_010248 | U34248 | Oliveros virus segment S | | Arenaviridae |
| ssRNA- | NC_010249 | AY216502 | Allpahuayo virus segment L | | Arenaviridae |
| ssRNA- | NC_010250 | AY216514 | Oliveros virus segment L | | Arenaviridae |
| ssRNA- | NC_010251 | AY216517 | Amapari virus segment L | | Arenaviridae |
| ssRNA- | NC_010252 | AY216519 | Cupixi virus segment L | | Arenaviridae |
| ssRNA- | NC_010253 | AY012687 | Allpahuayo virus segment S | | Arenaviridae |
| ssRNA- | NC_010254 | AF512832 | Cupixi virus segment S | | Arenaviridae |
| ssRNA- | NC_010255 | AY924390 | Bear Canyon virus segment L | | Arenaviridae |
| ssRNA- | NC_010256 | AY924391 | Bear Canyon virus segment S | | Arenaviridae |
| ssRNA- | NC_010562 | EU260463 | Chapare virus segment S | | Arenaviridae |
| ssRNA- | NC_010563 | EU260464 | Chapare virus segment L | | Arenaviridae |
| ssRNA- | NC_010700 | AF228063 | Whitewater Arroyo virus segment S | | Arenaviridae |
| ssRNA- | NC_010701 | AF485263 | Tamiami virus segment S | | Arenaviridae |
| ssRNA- | NC_010702 | AY924393 | Tamiami virus segment L | | Arenaviridae |
| ssRNA- | NC_010703 | AY924395 | Whitewater Arroyo virus segment L | | Arenaviridae |
| ssRNA- | NC_010756 | AF485261 | Parana virus segment S | | Arenaviridae |
| ssRNA- | NC_010757 | AF512831 | Flexal virus segment S | | Arenaviridae |
| ssRNA- | NC_010758 | AF512830 | Latino virus segment S | | Arenaviridae |
| ssRNA- | NC_010759 | EU627611 | Flexal virus segment L | | Arenaviridae |
| ssRNA- | NC_010760 | EU627612 | Latino virus segment L | | Arenaviridae |
| ssRNA- | NC_010761 | EU627613 | Parana virus segment L | | Arenaviridae |
| ssRNA- | NC_012702 | FJ554525 | Midway virus | Mononegavirales | Unclassified |
| ssRNA- | NC_012703 | FJ554526 | Nyamanini virus | Mononegavirales | Unclassified |
| ssRNA- | NC_013057 | EU914103 | Morogoro virus segment S | | Arenaviridae |
| ssRNA- | NC_013058 | EU914104 | Morogoro virus segment L | | Arenaviridae |
| ssRNA+ | NC_000940 | AF182760 | Porcine enteric calicivirus | | Caliciviridae |
| ssRNA+ | NC_000943 | AF161266 | Murray Valley encephalitis virus | | Flaviviridae |
| ssRNA+ | NC_001366 | X56019 | Theilovirus | Picornavirales | Picornaviridae |
| ssRNA+ | NC_001428 | D00538 | Human enterovirus C | Picornavirales | Picornaviridae |
| ssRNA+ | NC_001430 | D00820 | Human enterovirus D | Picornavirales | Picornaviridae |
| ssRNA+ | NC_001434 | L08816 | Hepatitis E virus | | HEV-like |
| ssRNA+ | NC_001437 | M18370 | Japanese encephalitis virus, genome | | Flaviviridae |
| ssRNA+ | NC_001449 | L04653 | Venezuelan equine encephalitis virus | | Togaviridae |
| ssRNA+ | NC_001461 | M31182 | Bovine viral diarrhea virus 1 | | Flaviviridae |
| ssRNA+ | NC_001472 | M16560 | Human enterovirus B | Picornavirales | Picornaviridae |
| ssRNA+ | NC_001474 | U87411 | Dengue virus type 2 | | Flaviviridae |
| ssRNA+ | NC_001475 | AY099336 | Dengue virus type 3 | | Flaviviridae |
| ssRNA+ | NC_001477 | U88536 | Dengue virus type 1 | | Flaviviridae |
| ssRNA+ | NC_001479 | M81861 | Encephalomyocarditis virus | Picornavirales | Picornaviridae |
| ssRNA+ | NC_001481 | L40021 | Feline calicivirus | | Caliciviridae |
| ssRNA+ | NC_001489 | M14707 | Hepatitis A virus | Picornavirales | Picornaviridae |
| ssRNA+ | NC_001490 | K02121 | Human rhinovirus B | Picornavirales | Picornaviridae |
| ssRNA+ | NC_001512 | M20303 | O'nyong-nyong virus | | Togaviridae |
| ssRNA+ | NC_001543 | M67473 | Rabbit hemorrhagic disease virus-FRG | | Caliciviridae |
| ssRNA+ | NC_001544 | M20162 | Ross River virus | | Togaviridae |
| ssRNA+ | NC_001545 | M15240 | Rubella virus | | Togaviridae |
| ssRNA+ | NC_001547 | J02363 | Sindbis virus | | Togaviridae |
| ssRNA+ | NC_001563 | M12294 | West Nile virus (lineage II strain 956) | | Flaviviridae |
| ssRNA+ | NC_001612 | U05876 | Human enterovirus A | Picornavirales | Picornaviridae |
| ssRNA+ | NC_001617 | A10937 | Human rhinovirus 89 | Picornavirales | Picornaviridae |
| ssRNA+ | NC_001639 | U15146 | Lactate dehydrogenase-elevating virus | | Arteriviridae |
| ssRNA+ | NC_001655 | AF179612 | Hepatitis GB virus B | | Flaviviridae |
| ssRNA+ | NC_001672 | U27495 | Tick-borne encephalitis virus | | Flaviviridae |
| ssRNA+ | NC_001710 | U44402 | GB virus C/Hepatitis G virus | | Flaviviridae |
| ssRNA+ | NC_001786 | U73745 | Barmah Forest virus | | Togaviridae |
| ssRNA+ | NC_001809 | Y07863 | Louping ill virus | | Flaviviridae |
| ssRNA+ | NC_001837 | U94421 | Hepatitis GB virus A | | Flaviviridae |
| ssRNA+ | NC_001846 | AF029248 | Murine hepatitis virus strain A59 | Nidovirales | Coronaviridae |
| ssRNA+ | NC_001859 | D00214 | Bovine enterovirus | Picornavirales | Picornaviridae |
| ssRNA+ | NC_001897 | AJ005695 | Human parechovirus, genome | Picornavirales | Picornaviridae |
| ssRNA+ | NC_001918 | AB010145 | Aichi virus | Picornavirales | Picornaviridae |
| ssRNA+ | NC_001943 | Z25771 | Human astrovirus | | Astroviridae |
| ssRNA+ | NC_001959 | M87661 | Norwalk virus | | Caliciviridae |
| ssRNA+ | NC_001961 | AF046869 | Porcine respiratory and reproductive syndrome virus | | Arteriviridae |
| ssRNA+ | NC_002031 | X03700 | Yellow fever virus | | Flaviviridae |
| ssRNA+ | NC_002032 | AF002227 | Bovine viral diarrhea virus genotype 2 | | Flaviviridae |
| ssRNA+ | NC_002058 | V01149 | Poliovirus | Picornavirales | Picornaviridae |
| ssRNA+ | NC_002306 | AJ271965 | Transmissible gastroenteritis virus | Nidovirales | Coronaviridae |
| ssRNA+ | NC_002469 | Y15937 | Ovine astrovirus | | Astroviridae |
| ssRNA+ | NC_002532 | X53459 | Equine arteritis virus | | Arteriviridae |
| ssRNA+ | NC_002551 | U76874 | Vesicular exanthema of swine virus | | Caliciviridae |
| ssRNA+ | NC_002554 | AF274010 | Foot-and-mouth disease virus - type C | Picornavirales | Picornaviridae |
| ssRNA+ | NC_002615 | Z69620 | European brown hare syndrome virus | | Caliciviridae |
| ssRNA+ | NC_002640 | AF326825 | Dengue virus type 4 | | Flaviviridae |
| ssRNA+ | NC_002645 | AF304460 | Human coronavirus 229E | Nidovirales | Coronaviridae |
| ssRNA+ | NC_002657 | AF326963 | Classical swine fever virus | | Flaviviridae |
| ssRNA+ | NC_003045 | AF391541 | Bovine coronavirus | Nidovirales | Coronaviridae |
| ssRNA+ | NC_003077 | AF361253 | Equine rhinovirus 3 | Picornavirales | Picornaviridae |
| ssRNA+ | NC_003092 | AF180391 | Simian hemorrhagic fever virus | | Arteriviridae |
| ssRNA+ | NC_003215 | X04129 | Semliki forest virus | | Togaviridae |
| ssRNA+ | NC_003417 | AF237947 | Mayaro virus | | Togaviridae |
| ssRNA+ | NC_003433 | AJ316246 | Sleeping disease virus | | Togaviridae |
| ssRNA+ | NC_003436 | AF353511 | Porcine epidemic diarrhea virus | Nidovirales | Coronaviridae |
| ssRNA+ | NC_003635 | AJ242984 | Modoc virus | | Flaviviridae |
| ssRNA+ | NC_003675 | AF144692 | Rio Bravo virus, genome | | Flaviviridae |
| ssRNA+ | NC_003676 | AF160193 | Apoi virus, genome |  | Flaviviridae |
| ssRNA+ | NC_003678 | AF144617 | Pestivirus Giraffe-1 | | Flaviviridae |
| ssRNA+ | NC_003679 | AF037405 | Border disease virus X818 | | Flaviviridae |
| ssRNA+ | NC_003687 | L06436 | Powassan virus | | Flaviviridae |
| ssRNA+ | NC_003690 | AF253419 | Langat virus | | Flaviviridae |
| ssRNA+ | NC_003899 | X63135 | Eastern equine encephalitis virus | | Togaviridae |
| ssRNA+ | NC_003900 | AF126284 | Aura virus | | Togaviridae |
| ssRNA+ | NC_003908 | AF214040 | Western equine encephalomyelitis virus | | Togaviridae |
| ssRNA+ | NC_003976 | AF327920 | Ljungan virus | Picornavirales | Picornaviridae |
| ssRNA+ | NC_003982 | X96870 | Equine rhinitis A virus | Picornavirales | Picornaviridae |
| ssRNA+ | NC_003983 | X96871 | Equine rhinitis B virus 1 | Picornavirales | Picornaviridae |
| ssRNA+ | NC_003985 | AJ011380 | Porcine teschovirus 1 | Picornavirales | Picornaviridae |
| ssRNA+ | NC_003987 | AF406813 | Porcine enterovirus A | Picornavirales | Picornaviridae |
| ssRNA+ | NC_003988 | AF201894 | Simian enterovirus A | Picornavirales | Picornaviridae |
| ssRNA+ | NC_003992 | AY593849 | Foot-and-mouth disease virus SAT 2 | Picornavirales | Picornaviridae |
| ssRNA+ | NC_003996 | AF285080 | Tamana bat virus, genome | | Flaviviridae |
| ssRNA+ | NC_004004 | AF308157 | Foot-and-mouth disease virus - type O | Picornavirales | Picornaviridae |
| ssRNA+ | NC_004064 | AY082891 | Calicivirus strain NB | | Caliciviridae |
| ssRNA+ | NC_004102 | AF009606 | Hepatitis C virus genotype 1 | | Flaviviridae |
| ssRNA+ | NC_004119 | AJ299445 | Montana myotis leukoencephalitis virus | | Flaviviridae |
| ssRNA+ | NC_004162 | AF369024 | Chikungunya virus | | Togaviridae |
| ssRNA+ | NC_004355 | AF331718 | Alkhurma virus | | Flaviviridae |
| ssRNA+ | NC_004421 | AB084788 | Bovine kobuvirus | Picornavirales | Picornaviridae |
| ssRNA+ | NC_004441 | AF363453 | Porcine enterovirus B | Picornavirales | Picornaviridae |
| ssRNA+ | NC_004451 | AY064708 | Simian picornavirus 1 | Picornavirales | Picornaviridae |
| ssRNA+ | NC_004541 | AF321298 | Walrus calicivirus | | Caliciviridae |
| ssRNA+ | NC_004542 | AB070225 | Canine calicivirus | | Caliciviridae |
| ssRNA+ | NC_004579 | AY179509 | Mink astrovirus | | Astroviridae |
| ssRNA+ | NC_004718 | AY274119 | SARS coronavirus | Nidovirales | Coronaviridae |
| ssRNA+ | NC_004915 | AY304994 | Foot-and-mouth disease virus Asia 1 | Picornavirales | Picornaviridae |
| ssRNA+ | NC_005039 | AB114858 | Yokose virus | | Flaviviridae |
| ssRNA+ | NC_005062 | AY193805 | Omsk hemorrhagic fever virus | | Flaviviridae |
| ssRNA+ | NC_005064 | AY149905 | Kamiti River virus | | Flaviviridae |
| ssRNA+ | NC_005147 | AY391777 | Human coronavirus OC43 | Nidovirales | Coronaviridae |
| ssRNA+ | NC_005831 | AY567487 | Human coronavirus NL63 | Nidovirales | Coronaviridae |
| ssRNA+ | NC_006269 | AY694184 | Sapovirus Hu/Dresden/pJG-Sap01/DE | | Caliciviridae |
| ssRNA+ | NC_006551 | AY453411 | Usutu virus | | Flaviviridae |
| ssRNA+ | NC_006554 | AY603425 | Sapovirus C12 strain C12 | | Caliciviridae |
| ssRNA+ | NC_006558 | AY702913 | Getah virus | | Togaviridae |
| ssRNA+ | NC_006577 | AY597011 | Human coronavirus HKU1 | Nidovirales | Coronaviridae |
| ssRNA+ | NC_006852 | MHV-JHM | Murine hepatitis virus strain JHM | Nidovirales | Coronaviridae |
| ssRNA+ | NC_006875 | AB117797 | Calicivirus isolate TCG | | Caliciviridae |
| ssRNA+ | NC_006947 | AY863002 | Karshi virus | | Flaviviridae |
| ssRNA+ | NC_007025 | DQ010921 | Feline coronavirus | Nidovirales | Coronaviridae |
| ssRNA+ | NC_007447 | AY427798 | Breda virus | Nidovirales | Coronaviridae |
| ssRNA+ | NC_007580 | DQ525916 | St. Louis encephalitis virus | | Flaviviridae |
| ssRNA+ | NC_007732 | DQ011855 | Porcine hemagglutinating encephalomyelitis virus | Nidovirales | Coronaviridae |
| ssRNA+ | NC_007916 | DQ013304 | Newbury agent 1 virus | | Caliciviridae |
| ssRNA+ | NC_008311 | DQ285629 | Murine norovirus 1 | | Caliciviridae |
| ssRNA+ | NC_008315 | DQ648794 | Bat coronavirus (BtCoV/133/2005) | Nidovirales | Coronaviridae |
| ssRNA+ | NC_008580 | AJ866991 | Rabbit vesivirus | | Caliciviridae |
| ssRNA+ | NC_008718 | DQ837641 | Entebbe bat virus | | Flaviviridae |
| ssRNA+ | NC_008719 | DQ837642 | Sepik virus | | Flaviviridae |
| ssRNA+ | NC_009019 | EF065505 | Bat coronavirus HKU4-1 | Nidovirales | Coronaviridae |
| ssRNA+ | NC_009020 | EF065509 | Bat coronavirus HKU5-1 | Nidovirales | Coronaviridae |
| ssRNA+ | NC_009021 | EF065513 | Bat coronavirus HKU9-1 | Nidovirales | Coronaviridae |
| ssRNA+ | NC_009026 | AY632536 | Bussuquara virus | | Flaviviridae |
| ssRNA+ | NC_009028 | AY632539 | Ilheus virus | | Flaviviridae |
| ssRNA+ | NC_009448 | EF165067 | Saffold virus | | Picornaviridae |
| ssRNA+ | NC_009657 | DQ648858 | Bat coronavirus 512/2005 | Nidovirales | Coronaviridae |
| ssRNA+ | NC_009693 | DQ071615 | Bat SARS CoV Rp3/2004 | Nidovirales | Coronaviridae |
| ssRNA+ | NC_009694 | DQ022305 | Bat coronavirus HKU3 | Nidovirales | Coronaviridae |
| ssRNA+ | NC_009695 | DQ412042 | Bat SARS CoV Rf1/2004 | Nidovirales | Coronaviridae |
| ssRNA+ | NC_009696 | DQ412043 | Bat SARS CoV Rm1/2004 | Nidovirales | Coronaviridae |
| ssRNA+ | NC_009823 | AF177036 | Hepatitis C virus genotype 2 | | Flaviviridae |
| ssRNA+ | NC_009824 | D17763 | Hepatitis C virus genotype 3, genome | | Flaviviridae |
| ssRNA+ | NC_009825 | Y11604 | Hepatitis C virus genotype 4, genome | | Flaviviridae |
| ssRNA+ | NC_009826 | Y13184 | Hepatitis C virus genotype 5, genome | | Flaviviridae |
| ssRNA+ | NC_009827 | D84262 | Hepatitis C virus genotype 6 | | Flaviviridae |
| ssRNA+ | NC_009887 | DQ902713 | Human enterovirus 100 | Picornavirales | Picornaviridae |
| ssRNA+ | NC_009891 | EU142040 | Seal picornavirus type 1 | Picornavirales | Picornaviridae |
| ssRNA+ | NC_009942 | DQ211652 | West Nile virus (lineage I strain NY99) | | Flaviviridae |
| ssRNA+ | NC_009988 | EF203064 | Bat coronavirus HKU2 | Nidovirales | Coronaviridae |
| ssRNA+ | NC_009996 | EF582385 | Human rhinovirus C | Picornavirales | Picornaviridae |
| ssRNA+ | NC_010327 | EF446615 | Equine coronavirus | Nidovirales | Coronaviridae |
| ssRNA+ | NC_010354 | EU236594 | Bovine rhinovirus 2 | Picornavirales | Picornaviridae |
| ssRNA+ | NC_010384 | AF414372 | Simian picornavirus strain N125 | Picornavirales | Picornaviridae |
| ssRNA+ | NC_010411 | AF326751 | Simian picornavirus 17 | Picornavirales | Picornaviridae |
| ssRNA+ | NC_010412 | AF326754 | Simian enterovirus SV19 | Picornavirales | Picornaviridae |
| ssRNA+ | NC_010413 | AF326761 | Simian enterovirus SV43 | Picornavirales | Picornaviridae |
| ssRNA+ | NC_010415 | AF326766 | Simian enterovirus SV6 | Picornavirales | Picornaviridae |
| ssRNA+ | NC_010436 | EU420137 | Bat coronavirus 1B | Nidovirales | Coronaviridae |
| ssRNA+ | NC_010437 | EU420138 | Bat coronavirus 1A | Nidovirales | Coronaviridae |
| ssRNA+ | NC_010438 | EU420139 | Bat coronavirus HKU8 | Nidovirales | Coronaviridae |
| ssRNA+ | NC_010624 | AY237420 | Sapovirus Mc10 | | Caliciviridae |
| ssRNA+ | NC_010646 | EU111742 | Coronavirus SW1 | Nidovirales | Coronaviridae |
| ssRNA+ | NC_010810 | EU376394 | Human TMEV-like cardiovirus | | Picornaviridae |
| ssRNA+ | NC_011050 | EF195384 | Steller sea lion vesivirus | | Caliciviridae |
| ssRNA+ | NC_011349 | DQ641257 | Seneca valley virus | Picornavirales | Picornaviridae |
| ssRNA+ | NC_011400 | FJ222451 | Astrovirus MLB1 | | Astroviridae |
| ssRNA+ | NC_011450 | AY593751 | Foot-and-mouth disease virus A | Picornavirales | Picornaviridae |
| ssRNA+ | NC_011451 | AY593838 | Foot-and-mouth disease virus SAT 1 | Picornavirales | Picornaviridae |
| ssRNA+ | NC_011452 | AY593850 | Foot-and-mouth disease virus SAT 3 | Picornavirales | Picornaviridae |
| ssRNA+ | NC_011548 | FJ376620 | Bulbul coronavirus HKU11-796 | Nidovirales | Coronaviridae |
| ssRNA+ | NC_011550 | FJ376622 | Munia coronavirus HKU13-3514 | Nidovirales | Coronaviridae |
| ssRNA+ | NC_011704 | EU871528 | Rabbit calicivirus Australia 1 MIC-07 | | Caliciviridae |
| ssRNA+ | NC_011829 | EU787450 | Porcine kobuvirus swine/S-1-HUN/2007/Hungary | Picornavirales | Picornaviridae |
| ssRNA+ | NC_012532 | AY632535 | Zika virus | | Flaviviridae |
| ssRNA+ | NC_012533 | AY632540 | Kedougou virus | | Flaviviridae |
| ssRNA+ | NC_012534 | AY632545 | Bagaza virus | | Flaviviridae |
| ssRNA+ | NC_012561 | FJ827631 | Highlands J virus | | Togaviridae |
| ssRNA+ | NC_012671 | FJ644291 | Quang Binh virus | | Flaviviridae |
| ssRNA+ | NC_012699 | FJ355928 | Calicivirus pig/AB90/CAN | | Caliciviridae |
| ssRNA+ | NC_012735 | EU707555 | Wesselsbron virus | | Flaviviridae |
| ssRNA+ | NC_012776 | FJ952384 | Lujo virus segment S | | Arenaviridae |
| ssRNA+ | NC_012777 | FJ952385 | Lujo virus segment L | | Arenaviridae |
| ssRNA+ | NC_012798 | FJ555055 | Human cosavirus E1 | Picornavirales | Picornaviridae |
| ssRNA+ | NC_012800 | FJ438902 | Human cosavirus A1 | Picornavirales | Picornaviridae |
| ssRNA+ | NC_012801 | FJ438907 | Human cosavirus B1 | Picornavirales | Picornaviridae |
| ssRNA+ | NC_012802 | FJ438908 | Human cosavirus D1 | Picornavirales | Picornaviridae |
| ssRNA+ | NC_012812 | FJ040215 | Bovine viral diarrhea virus 3 Th/04_KhonKaen | | Flaviviridae |
| ssRNA+ | NC_012936 | FJ938068 | Rat coronavirus Parker | Nidovirales | Coronaviridae |
| ssRNA+ | NC_012937 | FJ938051 | Feline coronavirus RM | Nidovirales | Coronaviridae |
| ssRNA+ | NC_012938 | FJ938053 | Feline coronavirus UU7 | Nidovirales | Coronaviridae |
| ssRNA+ | NC_012939 | FJ938054 | Feline coronavirus UU4 | Nidovirales | Coronaviridae |
| ssRNA+ | NC_012940 | FJ938056 | Feline coronavirus UU5 | Nidovirales | Coronaviridae |
| ssRNA+ | NC_012941 | FJ938060 | Feline coronavirus UU2 | Nidovirales | Coronaviridae |
| ssRNA+ | NC_012942 | FJ938061 | Feline coronavirus UU3 | Nidovirales | Coronaviridae |
| ssRNA+ | NC_012948 | FJ938065 | Bovine respiratory coronavirus AH187 | Nidovirales | Coronaviridae |
| ssRNA+ | NC_012949 | FJ938066 | Bovine respiratory coronavirus bovine/US/OH-440-TC/1996 | Nidovirales | Coronaviridae |
| ssRNA+ | NC_012950 | FJ938067 | Human enteric coronavirus strain 4408 | Nidovirales | Coronaviridae |
| ssRNA+ | NC_012951 | FJ938052 | Feline coronavirus UU11 | Nidovirales | Coronaviridae |
| ssRNA+ | NC_012952 | FJ938055 | Feline coronavirus UU8 | Nidovirales | Coronaviridae |
| ssRNA+ | NC_012953 | FJ938057 | Feline coronavirus UU15 | Nidovirales | Coronaviridae |
| ssRNA+ | NC_012954 | FJ938058 | Feline coronavirus UU16 | Nidovirales | Coronaviridae |
| ssRNA+ | NC_012955 | FJ938059 | Feline coronavirus UU10 | Nidovirales | Coronaviridae |
| ssRNA+ | NC_012956 | FJ938062 | Feline coronavirus UU9 | Nidovirales | Coronaviridae |
| ssRNA+ | NC_012957 | GQ179640 | Salivirus NG-J1 | Picornavirales | Picornaviridae |
| ssRNA+ | NC_012986 | GQ184145 | Human klassevirus 1 | Picornavirales | Picornaviridae |
| ssRNA+ | NC_013060 | FJ973620 | Astrovirus VA1 | | Astroviridae |
| ssRNA+ | NC_013114 | AB426608 | Human enterovirus 98 | Picornavirales | Picornaviridae |
